# Spatiotemporal regulation of Aurora B recruitment ensures release of cohesion during *C. elegans* oocyte meiosis

**DOI:** 10.1038/s41467-018-03229-5

**Published:** 2018-02-26

**Authors:** Nuria Ferrandiz, Consuelo Barroso, Oana Telecan, Nan Shao, Hyun-Min Kim, Sarah Testori, Peter Faull, Pedro Cutillas, Ambrosius P. Snijders, Monica P. Colaiácovo, Enrique Martinez-Perez

**Affiliations:** 1 MRC London Institute of Medical Sciences, Imperial College London, London, W12 0NN UK; 2 Department of Genetics, Harvard Medical School, Boston, MA 02115 USA; 3 Present Address: Barts Cancer Institute, London, EC1M 6BQ, UK; 4 Present Address: The Francis Crick Institute, London, NW1 1AT, UK; 5 Present Address: SPST, Tianjin University, Tianjin, 300072, China

## Abstract

The formation of haploid gametes from diploid germ cells requires the regulated two-step release of sister chromatid cohesion (SCC) during the meiotic divisions. Here, we show that phosphorylation of cohesin subunit REC-8 by Aurora B promotes SCC release at anaphase I onset in *C. elegans* oocytes. Aurora B loading to chromatin displaying Haspin-mediated H3 T3 phosphorylation induces spatially restricted REC-8 phosphorylation, preventing full SCC release during anaphase I. H3 T3 phosphorylation is locally antagonized by protein phosphatase 1, which is recruited to chromosomes by HTP-1/2 and LAB-1. Mutating the N terminus of HTP-1 causes ectopic H3 T3 phosphorylation, triggering precocious SCC release without impairing earlier HTP-1 roles in homolog pairing and recombination. CDK-1 exerts temporal regulation of Aurora B recruitment, coupling REC-8 phosphorylation to oocyte maturation. Our findings elucidate a complex regulatory network that uses chromosome axis components, H3 T3 phosphorylation, and cell cycle regulators to ensure accurate chromosome segregation during oogenesis.

## Abstract (web summary)

During meiosis, step-wise release of sister chromatid cohesion mediated by REC-8 cohesin is required for the formation of haploid gametes. Here, the authors show that in *C. elegans* oocytes, regulated recruitment of Aurora B kinase ensures the correct distribution of REC-8 phosphorylation, which promotes cohesion release.

## Introduction

Ensuring that daughter cells receive the correct number of chromosomes during cell division is essential for genome stability. By mediating sister chromatid cohesion (SCC) between S phase and anaphase, the cohesin complex plays a central role in this process. The core cohesin complex, consisting of two SMC proteins (Smc1 and Smc3) and the Scc1 kleisin, forms a tripartite ring-like structure that topologically entraps sister chromatids, thereby providing SCC^1^. At the onset of anaphase, cleavage of Scc1 by the protease separase releases cohesin’s embrace of sister chromatids, allowing their disjunction to opposite poles of the spindle. Precocious release of SCC causes severe defects in chromosome segregation, therefore cohesin cleavage must be tightly regulated. This is largely achieved by controlling the activation of the anaphase-promoting complex (APC), which degrades the separase inhibitor securin, and by phosphorylation events on Scc1 that greatly enhance its cleavability by separase. Thus, kinases that mediate Scc1 phosphorylation play an important role in promoting chromosome segregation^2–4^.

During meiosis, a single round of DNA replication is followed by two rounds of chromosome segregation to create haploid gametes from diploid germ cells, and errors in this process lead to sterility and the formation of aneuploid gametes^5^. Accurate chromosome segregation during meiosis depends on the creation and orderly dissolution of physical connections between sister chromatids and homologous chromosomes (homologs)^6^. First, sister chromatids are tethered by meiosis-specific cohesin complexes containing the kleisin subunit Rec8 instead of Scc1. Second, meiotic recombination leads to the formation of inter-homolog crossover events, which together with SCC provide the basis of chiasmata: attachments that hold the four sister chromatids of the homologs together, forming bivalent chromosomes. Third, sister kinetochores are mono-oriented on the first metaphase plate, while maternal and paternal kinetochores attach to microtubules from opposite sides of the spindle. Fourth, at the onset of anaphase I cohesin around centromeric regions is protected from separase cleavage, while cohesin in chromosome arms is cleaved, inducing segregation of homologs to opposite poles of the meiosis I spindle. Finally, sister kinetochores are bioriented on the metaphase II plate and cohesin cleavage promotes segregation of sister chromatids, producing haploid cells.

Precocious loss of cohesion is thought to be a major contributor to aneuploidy in oocytes^7^, but the molecular events that determine the pool of cohesin that separase must remove at anaphase I onset in oocytes have not been revealed. In yeast, Rec8 phosphorylation promotes separase cleavage during the meiotic divisions and the protection of centromeric cohesin relies on a mechanism that recruits the protein phosphatase 2A (PP2A), which is thought to antagonize Rec8 phosphorylation^8–12^. In mouse oocytes, separase activation triggers chiasma resolution and PP2A is required for protection of centromeric cohesin during meiosis I^13,14^. Similarly, separase is required for accurate chromosome partitioning in *Caenorhabditis elegans* oocytes and protein phosphatase 1 (PP1) is required to prevent precocious loss of cohesion during anaphase I^15–17^. However, the functional targets of PP2A and PP1 during oocyte meiosis have not been identified. Moreover, whether Rec8 is phosphorylated to promote cohesin removal in oocytes, and, if so, how this process is regulated, is not known.

In addition to meiosis-specific cohesin, conserved HORMA-domain proteins also associate with chromosome axes to promote homolog pairing, crossover formation, and checkpoint control during meiotic prophase. HORMA-domain proteins HTP-1 and HTP-2 in *C. elegans* are also required to prevent precocious release of SCC during the first meiotic division^18,19^, and mouse oocytes lacking HORMAD1 display defects in SCC after the first meiotic division^20^. Thus, HORMA-domain proteins play important, but poorly understood, roles in SCC protection during the first meiotic division.

Here, we demonstrate that REC-8 is phosphorylated by Aurora B to promote release of SCC during the first meiotic division in *C. elegans* oocytes. Correct distribution of REC-8 phosphorylation on metaphase I bivalents is achieved by regulating the recruitment of Aurora B to chromatin. HORMA-domain proteins HTP-1/2 act as local antagonists of H3 pT3, a histone mark dependent on the Haspin kinase that promotes Aurora B recruitment, while CDK-1 temporally couples Aurora B recruitment to oocyte maturation. Our results reveal the molecular mechanisms that control the two-step release of cohesion during oogenesis.

## Results

### AIR-2 phosphorylates REC-8 during late meiotic prophase

**Fig. 1:**
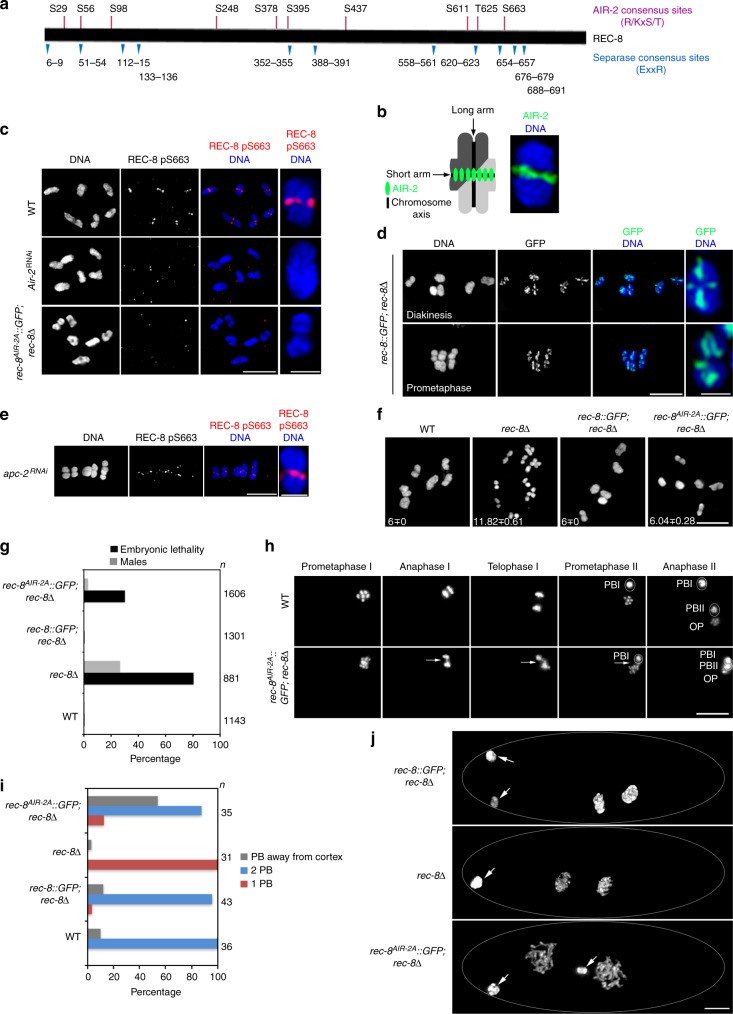
AIR-2 phosphorylates REC-8 to promote SCC release. **a** Diagram of REC-8 protein indicating the position of consensus motifs for AIR-2 phosphorylation (purple) and separase (blue). **b** Schematic representation of a single late diakinesis bivalent indicating the short arm bound by AIR-2 and the long arm lacking AIR-2. Projection of a diakinesis bivalent stained with anti-AIR-2 antibodies and DAPI. Note that AIR-2 associates exclusively with the short arm. **c** Projections of diakinesis oocytes stained with anti-REC-8 pS663 antibodies and DAPI. REC-8 pS663 signal is only found in the short arm of WT bivalents, and the signal is absent following depletion of *air-2* and in *rec-8* ^*AIR-2A*^ mutants carrying the S663A substitution. A single bivalent is magnified in the last column. **d** Projections of late diakinesis and prometaphase oocytes from worms expressing REC-8::GFP stained with anti-GFP antibodies and DAPI. GFP signal (REC-8) is observed in both the long and short arms. A single bivalent is magnified in the last column. **e** *apc-2* depleted oocyte arrested at metaphase I stained with anti-REC-8 pS663 antibodies and DAPI. REC-8 pS663 signal remains present in the short arms. A single bivalent is magnified in the last column. **f** Projections of diakinesis oocytes stained with DAPI, six bivalents are present in WT, *rec-8*::GFP; *rec-8∆* and *rec-8*^*AIR-2A*^::GFP; *rec-8∆*, while *rec-8∆* mutants show 12 bilobed univalents. Numbers indicate average number of DAPI-stained bodies per oocyte (at least 25 oocytes scored per genotype). **g** Percentage of embryonic lethality and incidence of male progeny in worms of indicated genotypes (*n* indicates total number of eggs counted). Note high levels of embryonic lethality in *rec-8*^*AIR-2A*^ mutants. **h** Selected frames from the embryos shown in Supplementary Movie 1 (WT) and Supplementary Movie 2 (*rec-8* ^*AIR-2A*^). PBI first polar body, PBII second polar body, OP oocyte pronucleus. Arrows point to chromatin bridges. **i** Quantification of the number and location of polar bodies (PB) in embryos of indicated genotypes (*n* indicates number of embryos scored). **j** Projections of DAPI-stained embryos at the two-cell stage. Arrows point to polar bodies. Scale bars = 5 μm in whole oocyte and embryo projections and 1 µm in panels containing a single magnified bivalent in **c**, **d**, **e**

Analysis of REC-8 amino acid sequence reveals the presence of 10 weak consensus sequences for Aurora B substrate (R/KXS/T), and that 7 of these sites are located in close proximity to the separase consensus motif (EXXR)^2,4,21^ (Fig. 1a). *C. elegans* Aurora B (AIR-2 from now on) is recruited to the short arm of late diakinesis bivalents, the region in which SCC will be released during anaphase I^15,17^(Fig. 1b), and can phosphorylate REC-8 in vitro at the Aurora B consensus site T625^15^. However, whether AIR-2 phosphorylates REC-8 in vivo and whether this event affects cohesin removal is not known. We raised phospho-specific antibodies against Aurora B consensus site S663 (Fig. 1a) and observed that REC-8 pS663 signal was only present in the short arm of the last two diakinesis oocytes (Fig. 1c), consistent with the temporal and spatial recruitment of AIR-2 to diakinesis bivalents. Knockdown of AIR-2 by RNA interference (RNAi) confirmed that pS663 signal is AIR-2 dependent (Fig. 1c and Supplementary Fig. 1A). Moreover, REC-8 pS663 signal was not observed in the long arm of wild-type diakinesis bivalents (Fig. 1c), despite extensive presence of REC-8 (Fig. 1d), or in diakinesis bivalents from transgenic worms expressing a mutant REC-8 in which S663 was mutated to alanine (see below) (Fig. 1c). These observations are consistent with REC-8 pS663 antibodies recognizing a pool of REC-8 that is phosphorylated by AIR-2.

If AIR-2 phosphorylates REC-8 to promote its cleavage by separase at anaphase onset, then phosphorylation of S663 should persist on the short arm of metaphase I bivalents. REC-8 is still present in both arms of prometaphase bivalents, although its intensity is often reduced in the short arm (Fig. 1d). Imaging of oocytes arrested at metaphase I, using knockdown of the APC, confirmed the presence of REC-8 pS663 in the short arm of bivalents (Fig. 1e).

### REC-8 phosphorylation promotes proper chromosome segregation

We tested the functional relevance of REC-8 phosphorylation by creating worms expressing a *rec-8*::*GFP* transgene (*rec-8*^*AIR-2A*^::*GFP*) in which the serine or threonine of 7 consensus sequences for Aurora B substrate were mutated to alanine (S56, S378, S395, S437, S611, T625, S663). To avoid potential redundancy, we also mutated to alanine one serine and two threonine residues (S396, T626, T662) located immediately adjacent to predicted AIR-2 phosphorylation sites. A transgene expressing wild-type REC-8::GFP rescued the meiotic defects and embryonic lethality of *rec-8* mutants^22,23^ (Fig. 1f, g). The REC-8^AIR-2A^::GFP protein localized to chromosomes (Supplementary Fig. 1B–C) and fully rescued the chiasma formation defect of *rec-8* mutants (Fig. 1f). However, elevated levels of embryonic lethality and male progeny among the progeny of *rec-8* ^*AIR-2A*^::*GFP* worms suggested abnormal chromosome segregation during the meiotic divisions (Fig. 1g). In vivo imaging of the meiotic divisions in *rec-8*^*AIR-2A*^ embryos revealed the presence of anaphase bridges, unequal partitioning of chromosomes, and failures in polar body extrusion (Fig. 1h and Supplementary Movies 1, 2). Oocyte meiosis results in the formation of a haploid oocyte pronucleus plus two polar bodies that are extruded away from the cytoplasm of the fertilized egg (Fig. 1j and Supplementary Movie 1). In contrast, 54% of *rec-8*^*AIR-2A*^ embryos displayed a centrally located polar body (Fig. 1i, j) and 12% produced a single polar body (Fig. 1i, j). These defects were notably different from those observed in *rec-8*-null mutants in which chiasmata are not formed, sister chromatids undergo equational separation during the first meiotic division^19^, and a single polar body is formed (Fig. 1i, j, Supplementary Movie 3, ref.^19^). Finally, knockdown of PP1 caused gross separation of sister chromatids during the first meiotic division of embryos expressing wild-type REC-8::GFP, as previously described in wild-type embryos^15,17^, but not in embryos expressing REC-8^AIR-2A^ (Supplementary Fig. 1D). The phenotypes observed in *rec-8*^*AIR-2A*^ mutants suggest that phosphorylation of REC-8 by AIR-2 is key for the timely release of SCC during the meiotic divisions.

### HTP-1 N terminus regulates meiotic chromosome segregation

**Fig. 2:**
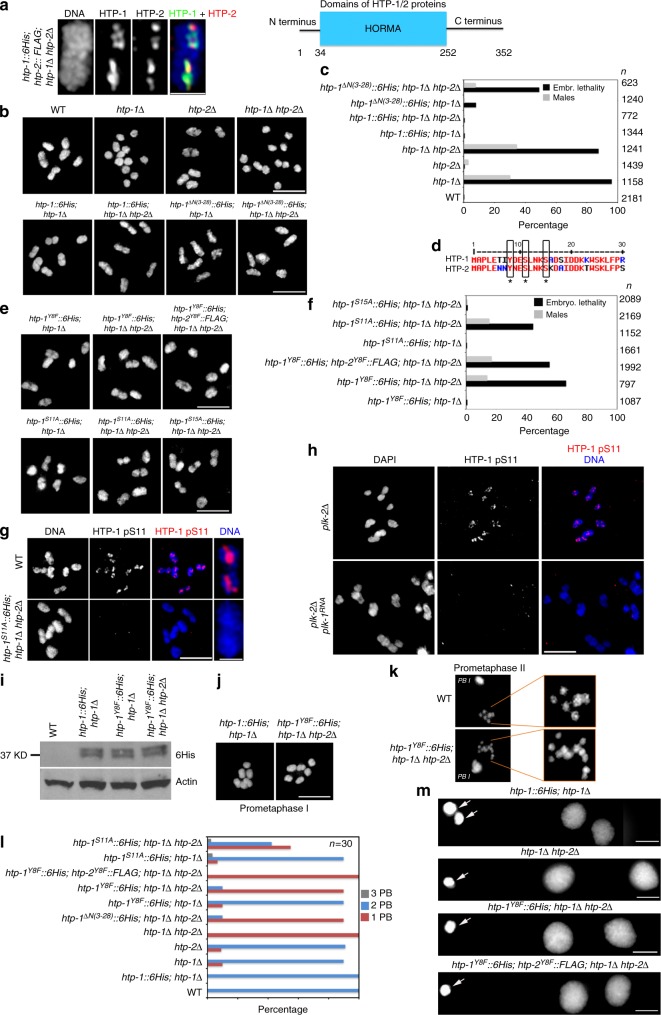
The N terminus of HTP-1/2 prevents premature cohesion release. **a** Diakinesis bivalent from worms expressing HTP-1::6His and HTP-2::FLAG stained with anti-6HIS and anti-FLAG antibodies and DAPI. HTP-1/2 are only detected in the long arm. Schematic representation of the HTP-1/2 proteins. **b** Projections of diakinesis oocytes stained with DAPI. Six DAPI-stained bodies (bivalents) indicate normal chiasma formation, while 12 DAPI-stained bodies (univalents) indicate a failure in chiasma formation. **c** Percentage of embryonic lethality and incidence of males in worms of indicated genotypes (*n* = number of eggs counted). Note high levels of embryonic lethality when HTP-1^ΔN(3-28)^ is expressed in a *htp1Δ htp-*2Δ double mutant background. **d** Diagram of the N terminus of HTP-1 and HTP-2 indicating the position of Y8, S11, and S15. **e** Projections of diakinesis oocytes of indicated genotypes stained with DAPI. **f** Percentage of embryonic lethality and incidence of males in worms of indicated genotype (*n* = number of eggs counted). Note high levels of embryonic lethality when HTP-1^Y8F^, HTP-1^S11A^ or HTP-1^Y8F^, and HTP-2^Y8F^ are expressed in a *htp1Δ htp-*2Δ double mutant background. **g**,**h** Projections of diakinesis oocytes stained with anti-HTP-1 pS11 antibodies and DAPI. HTP-1 pS11 staining is absent in *htp-1*^*S11A*^ mutant oocytes (**g**). HTP-1 pS11 signal is absent in oocytes from *plk-2(tm1395)*; *plk-1*^*RNAi*^ mutants, but not *plk-2(tm1395)* single mutants (**h**). **i** Western blot of whole-worm extracts probed with anti-6His antibodies. Note similar levels of HTP-1 expression in all genotypes except in WT controls lacking an *htp-1* transgene. **j** Projections of prometaphase I embryos stained with DAPI. Note the presence of 6 bivalents in control and *htp-1*^*Y8F*^; *htp1Δ htp-2Δ* mutant embryos. **k** Selected frames of prometaphase II embryos from the WT embryo shown in Supplementary Movie 1 and from the *htp-1*^*Y8F*^; *htp1Δ htp-2Δ* mutant embryo shown in Supplementary Movie 3. Six chromosomes are visible in the WT embryo, while the *htp-1*^*Y8F*^; *htp1Δ htp-2Δ* mutant embryo displays 12 chromatids. **l** Quantification of the number of polar bodies (PBs) in embryos of indicated genotypes (*n* = number of embryos scored). **m** Projections of DAPI-stained embryos of indicated genotypes at the two-cell stage. Arrows point to polar bodies. Scale bars = 5 μm in whole oocyte and embryo projections and 1 µm in panels containing a single magnified bivalent in **a**, **g**

If phosphorylation of REC-8 by AIR-2 regulates cohesin removal during the meiotic divisions, then chromosomal targeting of AIR-2 must be carefully regulated in diakinesis oocytes. Indeed, loss of PP1 or the PP1-interactor LAB-1 causes ectopic AIR-2 recruitment to the long arm of diakinesis bivalents and premature release of SCC during meiosis I^15,17,24^ (Supplementary Fig. 1D), but how PP1 and LAB-1 antagonize AIR-2 recruitment is not known. HORMA-domain proteins HTP-1 and HTP-2, which localize exclusively to the long arm of diakinesis bivalents (Fig. 2a) and form a complex in vivo with LAB-1, are also required to prevent premature loss of SCC during meiosis I^18,24,25^. HTP-1 and HTP-2 share 82% amino acid identity, and although only HTP-1 can ensure pairing and crossover formation during meiotic prophase, either HTP-1 or HTP-2 are sufficient to prevent premature loss of SCC during anaphase I^18,26^. Thus, we anticipated that mutations specifically affecting the role of HTP-1 in SCC release would only cause defects in chromosome segregation in the absence of HTP-2, and decided to focus on the N-terminus domain of HTP-1/2, the function of which is unknown (Fig. 2a). Expression of a HTP-1 protein lacking its N terminus (HTP-1^ΔN(3–28)^), which localized normally to pachytene chromosomes and to the long arm of diakinesis bivalents (Supplementary Fig. 2A), fully rescued chiasma formation in diakinesis oocytes of both *htp-1* single and *htp-1 htp-2* double mutants (Fig. 2b). Thus, HTP-1^ΔN(3–28)^ is competent in promoting pairing and recombination independently of HTP-2. However, we observed high levels of embryonic lethality and males among the progeny of worms expressing HTP-1^ΔN(3-28)^ in a *htp-1 htp-2* background, but not in a *htp-1* single mutant background (Fig. 2c), suggesting a role for the N terminus of HTP-1 in regulating meiotic chromosome segregation that is redundant with HTP-2.

### HTP-1 S11 and Y8 prevent precocious SCC loss at anaphase I

We used mass spectrometry to investigate if post-translational modifications on the N terminus of HTP-1 contribute to promote correct chromosome segregation. Phospho-enrichment experiments identified a peptide spanning residues 2–23 of HTP-1 that contained two phosphorylation events distributed between Y8, S11 and S15 (Supplementary Fig. 2B), three residues conserved between HTP-1 and HTP-2 (Fig. 2d). To investigate the functional relevance of these sites, we created transgenic worms expressing non-phosphorylatable versions of Y8 (Y8F), S11 (S11A), and S15 (S15A). The *htp-1*^*Y8F*^ and *htp-1*^*S11A*^ transgenes fully rescued defects in chiasma formation and embryonic lethality of *htp-1* single mutants, but caused high levels of embryonic lethality and male progeny when crossed into a *htp-1 htp-2* double mutant background, despite normal chiasma formation (Fig. 2e, f). In contrast, the *htp-1*^*S15A*^ transgene rescued embryonic viability in *htp-1 htp-2* double mutants, suggesting that S15 phosphorylation is not required for HTP-1 function (Fig. 2e, f). Raising phospho-specific antibodies against HTP-1 S11 we confirmed the presence of HTP-1 pS11 signal in the long arm of wild-type diakinesis oocytes (Fig. 2g and Supplementary Fig. 2C), and also that S11 phosphorylation depends on polo-like kinase 1 and 2 (PLK-1 and PLK-2) (Fig. 2h and Supplementary Fig. 2D–E), the *C. elegans* Polo-like kinase homologs that play key roles during meiosis^27,28^. This functional analysis suggests that Y8 and S11 on HTP-1 are required for fertility when HTP-2 is absent. In fact, transgenic worms carrying the Y8F mutation in both HTP-1 and HTP-2 showed high levels of embryonic lethality and male progeny despite normal chiasma formation (Fig. 2e, f). Importantly, levels of the HTP-1^Y8F^::6His protein are similar to those of the control wild-type HTP-1::6His (Fig. 2i and Supplementary Fig. 2F), which fully rescues viability of *htp-1 htp-2* mutants, confirming that viability defects are due to the Y8F mutation and not to differences in protein levels.

In vivo imaging of the meiotic divisions in worms expressing HTP-1^Y8F^::6His in a *htp-1 htp-2* background confirmed severe defects in chromosome segregation. While the completion of the first meiotic division in wild-type embryos produced a polar body plus a prometaphase II nucleus with 6 chromosomes (each one containing two attached sister chromatids), up to 12 chromatin bodies were detected in the prometaphase II nucleus of *htp-1*^*Y8F*^::*6His* (*htp-1*Δ *htp-2*Δ) embryos (Fig. 2j, k and Supplementary Movies 1 and 4). Imaging of fixed *htp-1*^*Y8F*^::*6His* (*htp-1*Δ *htp-2*Δ) embryos arrested at the end of anaphase I confirmed the presence of fully detached sister chromatids (Supplementary Fig. 2G). Thus, bivalents are dismantled into individual sister chromatids during anaphase I of *htp-1*^*Y8F*^::*6His* (*htp-1*Δ *htp-2*Δ) mutant embryos.

### *htp-1* N-terminus mutants extrude a single polar body

Filming of the meiotic divisions and imaging of fixed embryos also revealed that while wild-type embryos produced two polar bodies, *htp-1*^*Y8F*^::*6His* (*htp-1*Δ *htp-2*Δ) embryos produced a single polar body (Fig. 2l, m and Supplementary Movies 1 and 4). Moreover, most embryos in *htp-1*^*Y8F*^::*6His htp-2*^*Y8F*^::*FLAG* (*htp-1*Δ *htp-2*Δ), *htp-1*^*ΔN(3-28)*^::*6His* (*htp-1*Δ *htp-2*Δ), and *htp-1*^*S11A*^::*6His* (*htp-1*Δ *htp-2*Δ) mutants also formed a single polar body (Fig. 2l, m). Given the similar phenotypes observed in these mutants (*htp-1*^*Y8F*^, *htp-1*^*S11A*^, and *htp-1*^*ΔN(3-28)*^) we will collectively refer to them as *htp-1* N-terminus mutants. Interestingly, the first meiotic division in *rec-8* mutants also resulted in the formation of a polar body plus a prophase II nucleus containing detached sister chromatids that failed to undergo a further round of chromosome segregation, producing embryos with a single polar body (Supplementary Movies 3, 4, ref.^19^). Therefore, the extrusion of a single polar body in the embryos of *htp-1* N-terminus mutants may be a secondary consequence to the precocious loss of SCC during anaphase I.

### *htp-1* N-terminus mutants display ectopic AIR-2 recruitment

**Fig. 3:**
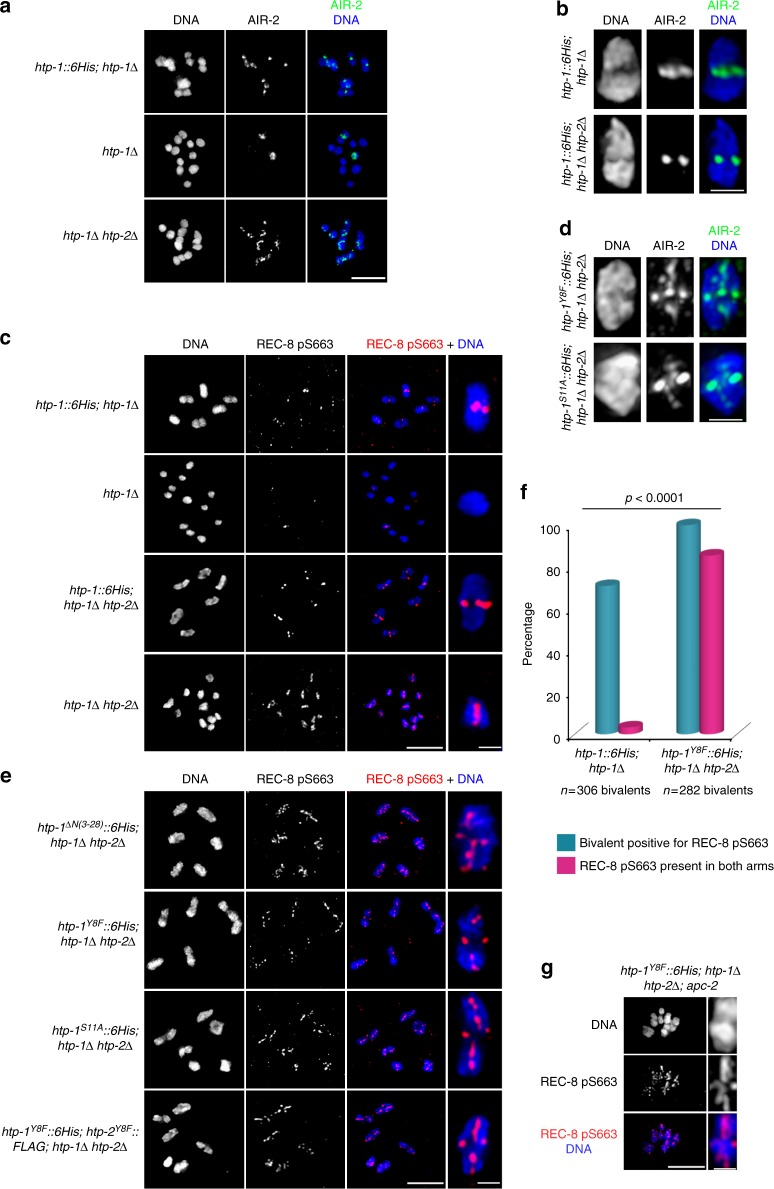
The N terminus of HTP-1/2 antagonizes AIR-2 recruitment. **a** Projections of diakinesis oocytes stained with anti-AIR-2 antibodies and DAPI. AIR-2 staining is detected in few univalents of *htp-1Δ* mutants, but in all univalents of *htp-1Δ htp-*2Δ mutants. **b**,**d** Projections of single diakinesis bivalents stained with anti-AIR-2 antibodies and DAPI. Note AIR-2 staining in the long arm when HTP-1^S11A^::6His or HTP-1^Y8F^::6His are expressed in a *htp1Δ htp-*2Δ double mutant background. **c**,**e** Projections of diakinesis oocytes stained with anti-REC-8 pS663 antibodies and DAPI. REC-8 pS663 staining is detected in few univalents of *htp-1Δ* mutants, but in all univalents of *htp-1Δ htp-*2Δ mutants (**c**). REC-8 pS663 staining is detected in the long arm when HTP-1 N-terminus mutant proteins are expressed in a *htp1Δ htp-*2Δ mutant background (**e**). **f** Quantification of the number of diakinesis bivalents positive for REC-8 pS663 (blue bar) and of the number of bivalents displaying REC-8 pS663 staining in both the short and the long arms (pink bar). *p* < 0.0001 by chi-square test. **g** Projection of an *apc-2*-depleted oocyte arrested in metaphase I stained with anti-REC-8 pS663 antibodies and DAPI. REC-8 pS663 signal remains present in both the short and long arms. Scale bars = 5 μm in whole oocyte projections and 1 µm in panels containing a single magnified bivalent in **b**, **c**, **d**, **e**. Scale bar = 0.75 µm in single bivalent shown in **g**

We next sought to clarify if the precocious loss of cohesion in anaphase I of *htp-1* N-terminus mutants was due to ectopic AIR-2 activity in diakinesis bivalents. As expected, AIR-2 and REC-8 pS663 staining was only observed in the short arm of diakinesis bivalents in oocytes of *htp-1* and *htp-1 htp-2* double mutants expressing a WT *htp-1*::*6His* transgene (Fig. 3a–c). Due to the requirement of HTP-1 in pairing and recombination^26,29^, chiasma formation fails in both *htp-1* and *htp-1 htp-2* mutant oocytes, but while few univalents of *htp-1* mutant oocytes were stained by AIR-2 or REC-8 pS663, all univalents of *htp-1 htp-2* oocytes displayed AIR-2 and REC-8 pS663 staining (Fig. 3a, c). Thus, both HTP-1 and HTP-2 antagonize AIR-2 recruitment and REC-8 pS663 phosphorylation in diakinesis oocytes. This prompted us to investigate if *htp-1* N-terminus mutants, which show normal chiasma formation and proper localization of HTP-1 to the long arm of diakinesis bivalents, retained the ability to antagonize AIR-2 recruitment. Strikingly, AIR-2 and REC-8 pS663 staining were present in both the short and long arms of diakinesis bivalents of worms expressing the HTP-1^ΔN(3-28)^, HTP-1^Y8F^, HTP-1^S11A^, or both HTP-1^Y8F^ and HTP-2^Y8F^ in a *htp-1 htp-2* double mutant background (Fig. 3d–f). The presence of ICP-1, an additional component of the chromosome passenger complex (CPC), and of H3 S10 phosphorylation, a known target of AIR-2, confirmed functional recruitment of AIR-2 to the long arm of diakinesis bivalents in *htp-1*^*Y8F*^::*6His* (*htp-1*Δ *htp-2*Δ) and *htp-1*^*S11A*^::*6His* (*htp-1*Δ *htp-2*Δ) oocytes (Supplementary Fig. 3). Moreover, APC knockdown confirmed that REC-8 phosphorylation persisted in all bivalent regions of *htp-1*^*Y8F*^*::6His* (*htp-1*Δ *htp-2*Δ) arrested at metaphase I (Fig. 3g). Thus, mutations in the N terminus of HTP-1/2 abolish the ability of these proteins to antagonize AIR-2 recruitment and REC-8 phosphorylation in diakinesis oocytes.

### The N terminus of HTP-1/2 antagonizes H3 T3 phosphorylation

**Fig. 4:**
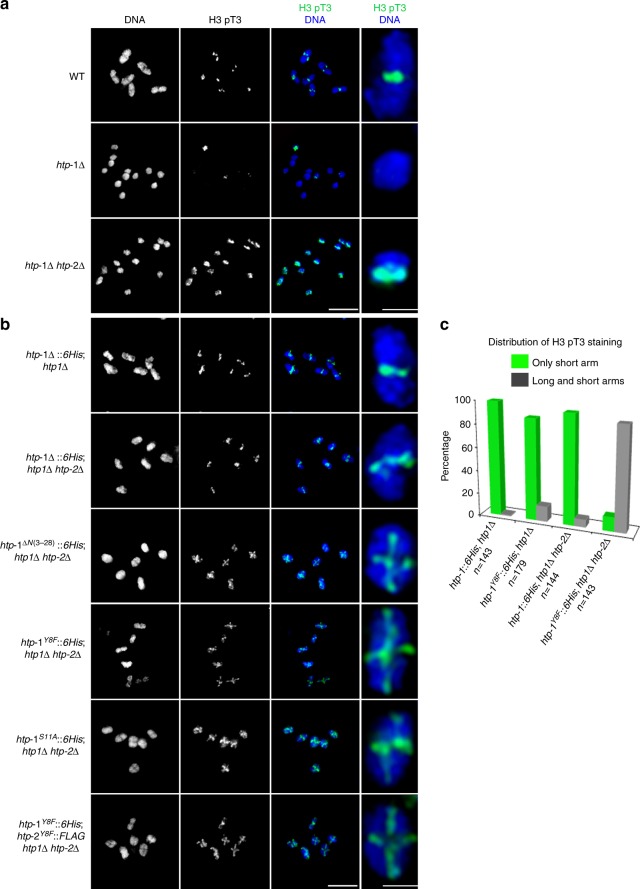
The N terminus of HTP-1/2 antagonizes H3 T3 phosphorylation. **a**,**b** Projections of diakinesis oocytes stained with anti-H3 pT3 antibodies and DAPI. H3 pT3 staining is detected in few univalents of *htp-1Δ* mutants, but in all univalents of *htp-1Δ htp-*2Δ mutants (**a**). H3 pT3 staining is detected in the long arm when HTP-1^ΔN(3-28)^, HTP-1^Y8F^, HTP-1^S11A^ or HTP-1^Y8F^, and HTP-2^Y8F^ are expressed in a *htp1Δ htp-*2Δ double mutant background. Scale bars = 5 μm in whole oocyte and embryo projections and 1 µm in panels containing a single magnified bivalent in **a**, **b**. **c** Quantification of the number of diakinesis bivalents displaying H3 pT3 staining only in the short arm (green) and in both the short and long arms (gray)

How AIR-2 is recruited to chromosomes during meiosis is not known, but in yeast and human mitotic cells the Survivin subunit of the CPC binds to chromatin containing histone 3 phosphorylated at threonine 3 to target Aurora B to centromeres^30–32^, prompting us to investigate H3 pT3 staining in diakinesis oocytes. H3 pT3 was only detected in the short arm of bivalents of wild type oocytes, in a few univalents of *htp-1* single mutants, and in all univalents of *htp-1 htp-2* double mutants, mimicking the localization of AIR-2 in those genotypes (Fig. 4a) and suggesting that HTP-1/2 could prevent AIR-2 recruitment by antagonizing H3 pT3. In agreement with this, H3 pT3 staining was observed in both the short and the long arms of diakinesis bivalents of worms expressing the HTP-1^ΔN(3-28)^, HTP-1^Y8F^, or HTP-1^S11A^ proteins in a *htp-1 htp-2* double mutant background (Fig. 4b, c). Ectopic phosphorylation of H3 T3 and REC-8 was also observed in bivalents of worms expressing a short truncation of HTP-1 removing Y8 and S11 (*htp-1*^*ΔN(6-13)*^*::6His*) in a *htp-1*Δ *htp-2*Δ background (Supplementary Fig. 4A). Therefore, the N terminus of HTP-1/2 acts to antagonize H3 T3 phosphorylation locally on diakinesis chromosomes.

### HTP-1/2 promote recruitment of PP1-interactor LAB-1

**Fig. 5:**
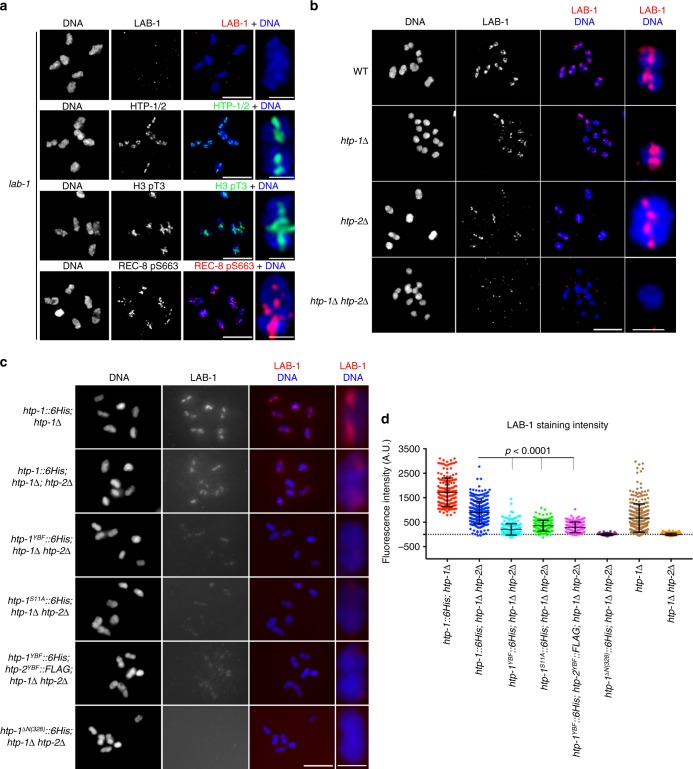
The N terminus of HTP-1/2 promotes LAB-1 recruitment. **a** Projections of diakinesis oocytes from *lab-1(tm1791)* mutants stained with anti-LAB-1, anti-HTP-1/2, anti-H3 pT3, and anti-REC-8 pS663 antibodies and DAPI. HTP-1/2 staining is limited to the long arms, but REC-8 pS663 and H3 pT3 signals are present in both the long and short arms. **b** Projections of diakinesis oocytes stained with anti-LAB-1 antibodies and DAPI. LAB-1 signal is absent in *htp1Δ htp-*2Δ double mutant oocytes. **c** Projections of diakinesis oocytes stained with anti-LAB-1 antibodies and DAPI. Data acquisition for all genotypes was performed using the same exposure settings and images shown are non-deconvolved projections adjusted with the same settings to allow visual comparisons of LAB-1 signal intensity. Note the reduction of LAB-1 signal when HTP-1^Y8F^, HTP-1^S11A^ or HTP-1^Y8F^, and HTP-2^Y8F^ are expressed in a *htp1Δ htp-*2Δ double mutant background, and the absence of LAB-1 signal when HTP-1^ΔN(3-28)^ is expressed in a *htp1Δ; htp-*2Δ double mutant background. **d** Quantification of LAB-1 fluorescence intensity in late diakinesis chromosomes. Between 84 and 192 bivalents and between 156 and 295 univalents were scored per genotype. Horizontal line indicates the mean and error bars indicate standard deviation of the mean; *p* < 0.0001 by *t*-test. Scale bars = 5 μm in whole oocyte projections and 1 µm in panels containing a single magnified bivalent in **a**, **b**, **c**

The precocious release of cohesion that we have observed on the first meiotic division of *htp-1* N-terminus mutants is highly reminiscent of the defects observed following PP1 knockdown and in worms lacking the PP1-interactor LAB-1^15,17,24^, which also display ectopic AIR-2 recruitment to the long arm of diakinesis bivalents^15,24^. Thus, we aimed to clarify the functional interplay between HTP-1/2, LAB-1, and PP1. *lab-1* mutant oocytes displayed ectopic H3 pT3 and REC-8 pS663 in the long arm of diakinesis bivalents, despite normal localization of HTP-1/2 (Fig. 5a), demonstrating that the ability of HTP-1/2 to antagonize H3 T3 phosphorylation requires LAB-1 and suggesting that HTP-1/2 may promote LAB-1 recruitment. Indeed, while LAB-1 was present in the long arm of *htp-2* mutant bivalents and in most univalents of *htp-1* mutants, LAB-1 was not detected in univalents of *htp-1 htp-2* double mutants, or in bivalents of worms expressing HTP-1^ΔN(3-28)^ in a *htp-1 htp-2* double mutant background (Fig. 5b–d). Although LAB-1 staining was detected in the long arm of diakinesis bivalents in *htp-1*^*Y8F*^::*6His* (*htp-1*Δ *htp-2*Δ), *htp-1*^*S11A*^::*6His* (*htp-1*Δ *htp-2*Δ), and *htp-1*^*Y8F*^::*6His htp-2*^*Y8F*^::*FLAG* (*htp-1*Δ *htp-2*Δ), the intensity of LAB-1 signal was reduced compared with worms expressing WT *htp-1*::*6His* (*htp-1*Δ *htp-2*Δ) (Fig. 5c, d). Oocytes lacking either HTP-1 or HTP-2 show a similar decrease in LAB-1 intensity compared with wild-type controls, confirming that both HTP-1 and HTP-2 promote LAB-1 recruitment in diakinesis oocytes. Interestingly, in all genotypes analyzed, the amount of chromosome-associated LAB-1 decreases in the diakinesis oocyte in the most proximal position of the germline (Supplementary Fig. 4B). These results demonstrate that the N terminus of HTP-1/2 promotes chromosomal recruitment of LAB-1 to antagonize H3 T3 phosphorylation.

### PP1 antagonizes H3 T3 phosphorylation in diakinesis oocytes

**Fig. 6:**
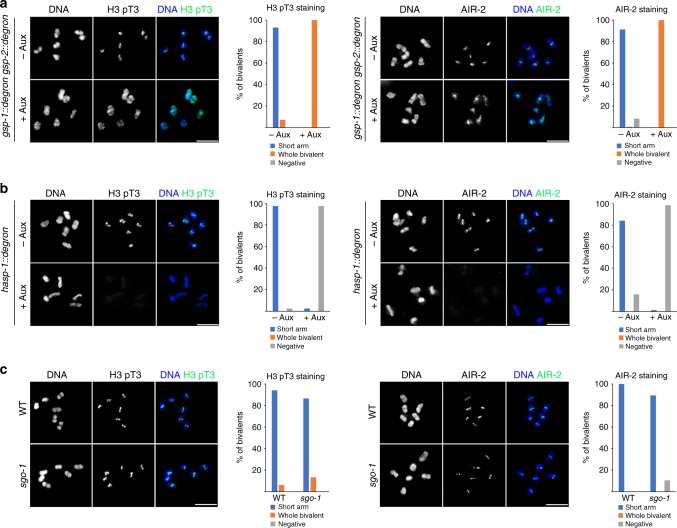
H3 T3 phosphorylation is mediated by Haspin and antagonized by PP1. Projections of late diakinesis oocytes stained with anti-H3 pT3 or anti-AIR-2 antibodies and DAPI. **a** Auxin-mediated depletion of PP1 (GSP-1 + GSP-2) causes spreading of H3 pT3 and AIR-2 staining to all bivalent regions. **b** Auxin-mediated depletion of HASP-1 causes undetectable H3 pT3 and AIR-2 staining in diakinesis oocytes. **c** *sgo-1* mutants display normal H3 pT3 and AIR-2 staining in the short arm of diakinesis bivalents. Between 19 and 35 late diakinesis oocytes (−1) were scored for quantifying H3 pT3 and AIR-2 staining in all genotypes. Scale bars = 5 μm

The ability of LAB-1 to antagonize AIR-2 recruitment depends on PP1^24^ and PP1 antagonizes H3 T3 phosphorylation in mammalian cells^33^. Thus, our results suggest that HTP-1/2 prevents AIR-2 recruitment by acting as a platform for loading LAB-1 and its interactor PP1, which ultimately could antagonize H3 T3 phosphorylation. To test this hypothesis, we first used CRISPR to mutate a PP1 docking site in LAB-1^25^, but the mutant LAB-1^KAIA^ protein failed to load on chromosomes (Supplementary Fig. 5A). Second, using CRISPR we tagged GSP-1 and GSP-2 (PP1 catalytic subunits beta and gamma) with an auxin-inducible degron sequence that allows rapid protein degradation in the *C. elegans* germline^34^. Following auxin treatment of the *gsp-1::degron gsp-2::degron* strain, both H3 pT3 and AIR-2 signals were observed on all regions of diakinesis bivalents, while H3 pT3 and AIR-2 were restricted to the short arm in untreated controls (Fig. 6a). Auxin-mediated depletion of GSP-1/2 did not affect HTP-1/2 localization to the long arm of diakinesis bivalents, and mutation of a putative PP1 docking site in the HORMA domain of HTP-1 did not induce defects during the meiotic divisions (Supplementary Fig. 5B, C). Therefore, PP1 is required to antagonize H3 pT3 on diakinesis oocytes and HTP-1/2 promote PP1 activity indirectly, via LAB-1 recruitment.

### Haspin is required for Aurora B recruitment in oocytes

In mitotic cells H3 T3 phosphorylation is mediated by Haspin^35^, which is recruited to chromosomes by the cohesin-associated protein Pds5^36,37^. The *C. elegans* genome contains two putative Haspin homologs, but only HASP-1 contains a conserved Pds5 interacting domain^37^. Depletion of HASP-1 in germlines of adult worms using the auxin degron system resulted in undetectable H3 pT3 staining in diakinesis oocytes, while oocytes from untreated controls displayed H3 pT3 in the short arm of diakinesis bivalents (Fig. 6b). Haspin depletion also resulted in undetectable AIR-2 staining (Fig. 6b), confirming that H3 pT3 promotes AIR-2 recruitment to diakinesis oocytes.

In yeast and mammals, centromeric recruitment of Aurora B is also promoted by Bub1-dependent phosphorylation of H2A T120, which in turn is recognized by the CPC interactor Shugoshin^30,38^. Since both BUB-1 and SGO-1 (Shugoshin) are recruited to the short arm of bivalents in late diakinesis oocytes^24,39^, we investigated if SGO-1 promotes chromosomal recruitment of the CPC in diakinesis oocytes by deleting the whole coding region of the *sgo-1* locus using CRISPR. Both H3 pT3 and AIR-2 localized normally to the short arm of diakinesis bivalents in *sgo-1* mutants (Fig. 6c). Thus, although Sgo1 promotes centromeric accumulation of H3 pT3 in mammalian cells^40^, H3 pT3 localization to the short arm of diakinesis bivalents in *C. elegans* occurs normally in the absence of SGO-1. We conclude that AIR-2 recruitment to diakinesis chromosomes requires the Haspin-H3 pT3 pathway, while the SGO-1 pathway alone is not sufficient for recruiting AIR-2 during late diakinesis.

### CDK-1 controls the timing of AIR-2 recruitment in oocytes

**Fig. 7:**
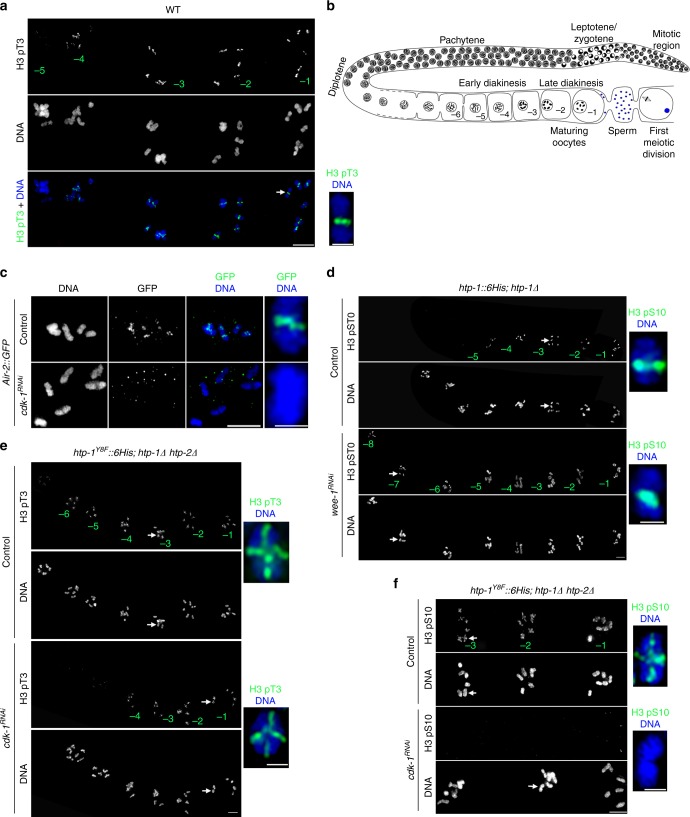
CDK-1 activation promotes AIR-2 recruitment. **a** Projection of diakinesis oocytes stained with anti-H3 pT3 antibodies and DAPI. The position of oocytes within the germline is indicated in green numbers. Inset shows a single bivalent from the −1 oocyte. **b** Diagram of a *C. elegans* germline indicating the temporal arrangement of diakinesis oocytes. The -1 diakinesis oocyte is about to be ovulated and undergo the first meiotic division. **c** Projections of diakinesis oocytes from worms expressing an *air-2*::*GFP* transgene stained with anti-GFP antibodies and DAPI. Depletion of *cdk-1* eliminates chromosomal GFP (AIR-2) staining. **d** Projections of *htp-1*::*6His; htp-1Δ* diakinesis oocytes stained with anti-H3 pS10 antibodies and DAPI. The position of oocytes within the germline is indicated in green numbers. Note that depletion of *wee-1* by RNAi anticipates H3 pS10 staining, which is visible in the −8 oocyte. **e**,**f** Projections of diakinesis oocytes from *htp-1*^*Y8F*^::*6His*; *htp1Δ htp-*2Δ mutants stained with anti-H3 pT3 (**d**) or anti-H3 pS10 (**e**) antibodies and DAPI. Note that depletion of *cdk-1* eliminates H3 pS10 staining in all oocytes, while H3 pT3 staining is eliminated from early diakinesis oocytes but persists in both arms of the −4, −3, −2, and −1 oocytes. Scale bars = 5 μm in whole oocyte projections and 1 µm in panels containing a single magnified bivalent (indicated by arrows in panel containing all oocytes) in **a**, **c**, **d**, **e**, **f**

Our results so far demonstrate that in diakinesis oocytes AIR-2 is recruited to chromosomal regions displaying H3 T3 phosphorylation. However, AIR-2 staining is only observed in the two more proximal oocytes (undergoing oocyte maturation), while H3 pT3 staining is present in immature (early diakinesis) oocytes (Fig. 7a, b), suggesting the existence of a temporal regulator of AIR-2 recruitment. The transition between diakinesis and metaphase I is mediated by the maturation promoting factor (MPF) containing CDK-1 and cyclin B^41^, and in *C. elegans* the signaling cascade that leads to MPF activation is initiated by major sperm proteins^42^. Since AIR-2 is not recruited to diakinesis chromosomes in worms lacking sperm^43^ and *cdk-1* knockdown reduces H3 S10 phosphorylation^44^, we investigated if AIR-2 recruitment is controlled by the MPF. RNAi of *cdk-1* prevented AIR-2 loading to the short arm of late diakinesis bivalents, despite the presence of H3 pT3 in these regions (Fig. 7c and Supplementary Fig. 6A). Interestingly, although *cdk-1* RNAi did not prevent H3 T3 phosphorylation in the most proximal oocytes, it reduced H3 pT3 signal in early diakinesis oocytes (Supplementary Fig. 6A). To confirm that AIR-2 activity is temporally controlled by MPF activity, we monitored the appearance of AIR-2-dependent H3 S10 phosphorylation following knockdown of the Wee1 kinase, which mediates inhibitory CDK phosphorylations that prevent MPF activity in immature oocytes^41^. *wee-1* RNAi induced H3 S10 and REC-8 S663 phosphorylation in early diakinesis oocytes (Fig. 7d and Supplementary Fig. 7A), confirming that MPF activity exerts temporal control of AIR-2 activity. Importantly, premature H3 pS10 and REC-8 pS663 staining in early diakinesis of *wee-1*^*RNAi*^ oocytes was limited to the short arm of bivalents, suggesting that MPF exerts temporal but not spatial control of AIR-2 activity. In agreement with this possibility, *cdk-1* RNAi did not prevent ectopic H3 T3 phosphorylation on the long arm of late diakinesis bivalents in *htp-1*^*Y8F*^*::6His* (*htp-1*Δ *htp-2*Δ) oocytes, but impaired H3 S10 and REC-8 S663 phosphorylation on all bivalent regions (Fig. 7e, f and Supplementary Fig. 6B). Moreover, *wee-1* RNAi in *htp-1*^*Y8F*^::*6His* (*htp-1*Δ *htp-2*Δ) mutants induced REC-8 pS663 staining in the long arm of early diakinesis oocytes (Supplementary Fig. 7B). These observations are consistent with H3 pT3 acting as a spatial cue for AIR-2 recruitment that is only implemented upon MPF activation during late diakinesis.

## Discussion

**Fig. 8:**
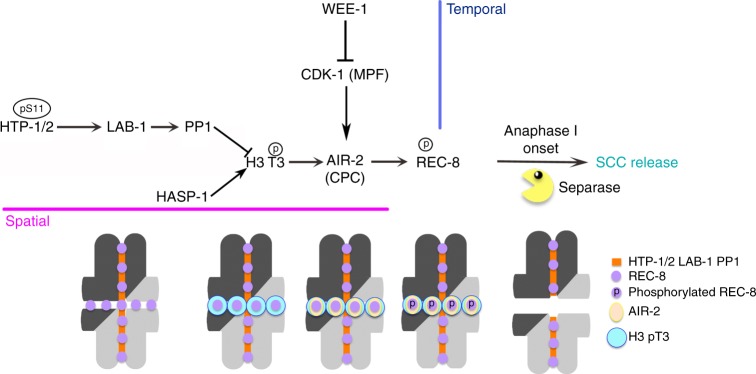
Model of the events that control the release of SCC in *C. elegans* oocytes. H3 T3 phosphorylation mediated by Haspin and antagonized by PP1 regulates the spatial recruitment of AIR-2 to diakinesis bivalents, while CDK-1 exerts temporal control. See the beginning of discussion for a detailed description of the main events depicted in the model. Bivalents shown at the bottom of the model indicate the sequential recruitment of different proteins and phosphorylation events between late diakinesis and the onset of anaphase I. Abbreviations: MPF (Maturation Promoting Complex), CPC (Chromosome Passenger Complex), encircled “p” indicates phosphorylation event

The stepwise removal of cohesin during the meiotic divisions is an essential aspect of sexual reproduction, ultimately enabling the formation of haploid gametes from diploid germ cells. We have elucidated the molecular mechanisms that control this process in *C. elegans* oocytes, demonstrating that recruitment of AIR-2 kinase to late diakinesis bivalents induces local phosphorylation of REC-8, marking these cohesin complexes for removal at the onset of anaphase I (Fig. 8). Spatially restricting AIR-2 recruitment before the onset of anaphase I is essential to prevent unscheduled removal of the subset of cohesin responsible for mediating cohesion until the second meiotic division. Haspin-dependent H3 T3 phosphorylation promotes chromosomal targeting of AIR-2 during late diakinesis and this histone mark is locally antagonized by HORMA-domain proteins HTP-1/2, which via LAB-1 promote chromosomal recruitment of PP1. Temporal regulation of AIR-2 recruitment is exerted by the activation of CDK-1 during oocyte maturation. These findings uncover a complex regulatory network that implements spatiotemporal control of AIR-2 recruitment and REC-8 phosphorylation to promote accurate chromosome segregation.

Although precocious loss of cohesion during the first meiotic division is thought to be a major contributor to aneuploidy in oocytes, the molecular events that specify the pool of cohesin that should be removed by separase at anaphase I onset during oogenesis had not been revealed. We have shown that the spatial distribution of AIR-2-dependent REC-8 phosphorylation in diakinesis bivalents determines the pattern of cohesion release during anaphase I. While REC-8 phosphorylation is restricted to the short arm of diakinesis bivalents in wild-type oocytes, which undergo two-step release of cohesion, in oocytes of *htp-1* N-terminus mutants both AIR-2 and phosphorylated REC-8 spread to the long arm of diakinesis bivalents and full release of cohesion occurs during anaphase I. Precocious break down of bivalents into individual sister chromatids during the first meiotic division also occurs in mouse oocytes lacking Shugoshin-2 or its interactor PP2A^14,45,46^, although the targets of PP2A during the first meiotic division remain unknown. Our studies are consistent with REC-8 phosphorylation marking the chromosomal regions in which cohesin is cleaved by separase at the onset of anaphase I to promote cohesion release, suggesting that this is a feature of meiosis conserved from yeast to higher eukaryotes. Whether Rec8 is phosphorylated in vivo in mammals to promote cohesion release remains unknown, but in vitro phosphorylation of Rec8 by Plk1 increases its cleavability by Separase^47^. Interestingly, mouse Rec8 carries 10 Aurora B consensus motifs and some of these sites occur in close proximity to consensus separase cleavage motifs (Supplementary Fig. 8). Future studies should address if Rec8 phosphorylation contributes to meiotic chromosome segregation in mammals.

Our studies reveal that the correct pattern of REC-8 phosphorylation in metaphase I bivalents of *C. elegans* oocytes is achieved by the tight spatiotemporal regulation of AIR-2 recruitment to chromosomes during late meiotic prophase. This contrasts with the strategy used in yeast, which relies on the targeted recruitment of PP2A to antagonize Rec8 phosphorylation at centromeres^8,9^. Haspin-dependent H3 pT3 is the spatial cue that induces AIR-2 recruitment to diakinesis chromosomes. Interestingly, while Haspin-mediated recruitment of AIR-2 to chromosomes in *C. elegans* oocytes ultimately promotes cohesin removal at anaphase I, Haspin protects cohesin from removal in mammalian cells^36,40^. This role reversal is likely related to the presence of holocentric (lacking a localized centromere) chromosomes in *C. elegans*, in which the position of the single crossover, rather than the centromere, dictates the chromosomal regions in which SCC must be protected during anaphase I^48^. In contrast to centromeres, crossovers do not occur at a predetermined position, and thus the machinery that promotes SCC release during meiosis in *C. elegans* must be flexible in terms of its spatial deployment to chromosomes. Since the CPC containing Aurora B is recruited to chromatin regions containing H3 pT3, the ability to control the spatial distribution of this histone mark by factors such as HTP-1/2, the localization of which is modulated by crossovers^18^, probably played an important role in establishing AIR-2 as the REC-8 kinase in *C. elegans.*

In addition to H3 T3 phosphorylation, chromosomal targeting of AIR-2 in oocytes also requires CDK-1 (as part of the maturation promoting factor). This is likely explained by the direct phosphorylation of a CPC component by CDK-1, as observed in mitotic cells^49^. Coupling REC-8 phosphorylation to the maturation process that releases oocytes from their developmental arrest before the first meiotic division may promote correct chromosome segregation in two ways. First, if REC-8 phosphorylation occurred during early prophase in oocytes, as observed in *Saccharomyces cerevisiae* and *Schizosaccharomyces pombe*^8,9^, unscheduled loss of phosphorylation events in just a small fraction of REC-8 complexes during oocyte arrest could interfere with cohesin removal at anaphase I onset. Second, entering prophase arrest with phosphorylated REC-8 would render oocytes highly susceptible to leaky activation of separase before maturation, which would result in premature cohesin removal. We suggest that temporal regulation of the phosphorylation events that promote separase cleavage of REC-8 at anaphase onset is a crucial aspect of oocyte meiosis. This may be highly relevant to human oocytes, which arrest for decades before undergoing oocyte maturation.

While the role of HORMA-domain proteins in promoting pairing, recombination, and checkpoint control during meiotic prophase is well documented, their role as protectors of SCC during the first meiotic division was not understood. We have shown that HTP-1/2 protect SCC by creating a scaffold (via LAB-1) that recruits PP1 to chromosomes to dephosphorylate T3 on histone H3, thus antagonizing AIR-2 recruitment. Notably, this role of HTP-1/2/LAB-1 appears analogous to that of Repo-Man in mammalian mitotic cells: targeting PP1 to chromatin to dephosphorylate T3 on H3, thus acting as a local antagonist of Aurora B recruitment^33,50^. The finding that the N terminus of HTP-1 promotes recruitment of LAB-1/PP1 to chromosomes, while being dispensable for earlier roles of HTP-1 in pairing and recombination, suggests functional specialization of this HTP-1 domain. A similar example is seen on the C terminus of *C. elegans* HORMA-domain proteins HIM-3 and HTP-3, which contain sites required for the chromosomal targeting of HTP-1/2^51^. In contrast, the best understood HORMA-domain protein, the spindle assembly checkpoint component Mad2, lacks N- or C-terminal domains and its HORMA domain mediates all known protein–protein interactions^52^. We propose that the acquisition of N- and C-terminal regions has endowed meiotic HORMA-domain proteins with the plasticity required to recruit a range of interactors that execute highly diverse meiotic tasks, such as promoting homolog pairing during early prophase and protecting SCC during the first meiotic division in the case of HTP-1. Our findings also suggest that post-translational modifications on HORMA-domain proteins can regulate protein recruitment to chromosomes, allowing dynamic modification of the molecular composition of chromosome axes.

Mammalian HORMAD1 and HORMAD2 share a similar organization with HTP-1/2 including a short N-terminal domain and a longer (120 residues) C-terminal domain, both of unknown function. Although HORMAD1/2 are best known for their roles in mediating paring and checkpoint control during early prophase, in metaphase I spermatocytes HORMAD1 localizes to centromeres^53^, the region in which SCC must be protected during anaphase I. Moreover, metaphase II oocytes in *Hormad1* mutant oocytes display defects in cohesion^20^, suggesting that similar to HTP-1/2, HORMAD1 may also play some role in regulating SCC during the meiotic divisions. Elucidating how the different regions of meiotic HORMA-domain proteins provide functionality of this group of versatile proteins remains an important goal for future studies.

## Methods

### *C. elegans* genetics

Unless indicated, all strains were maintained at 20 °C under standard conditions. N2 Bristol was used as wild-type strain. The following mutant alleles were used: LG I: *lab-1 (tm1791)*, *plk-2(tm1395)*, *spe-9 (hc88)*; LG III: *unc-119(ed3)*; LG IV: *htp-1 (gk174)*, *htp-2 (tm2543)*, *rec-8 (ok978)*. Note that for simplicity the *htp-1 (gk174)* and *htp-2 (tm2543)* alleles are referred to as *htp-1*Δ and *htp-2*Δ respectively. Supplementary Tables 1 and 2 contain a full list of the transgenes and strains used in this study.

### Creation of transgenic *C. elegans* strains

Transgenic worms were generated by single-copy insertions of the desired transgene in the *ttTi5605* locus on chromosome II or in the *oxTi177* locus on chromosome IV following the protocol described in ref. ^54^. The *rec-8::GFP* transgene carried 536 bp upstream of the starting codon, the entire sequence of the *rec-8* locus, a green fluorescent protein (GFP) complementary DNA containing 3 artificial introns and 665 bp of downstream sequence. The *htp-1*::*6His* transgene carried 620 bp upstream of the starting codon, the entire sequence of *htp-1* locus, a 6His tag, and 183 bp of downstream sequence. The *htp-2*::*FLAG* transgene carried 482 bp upstream of starting codon, the entire sequence of *htp-2* locus, a FLAG tag, and 318 bp of downstream sequence. The *lab-1*^*KAIA*^ mutant was created by CRISPR with a single-guide RNA targeting the ‘atcttcgaacatcgttccgg’ sequence at the 3’ end of *lab-1* and this sequence was cloned into pHKMC1 (Addgene plasmid number 67720) as described in ref. ^55^. For the repair template a sequence ~1 KB (upstream and downstream sequence from the site of the double-strand break (DSB)) was cloned into pCRbluntII topo vector including the mutations for KVIW to KAIA. Expression of sgRNA Cas9 was performed using the protocols described in ref. ^56^.

Insertion of a 135 bp sequence, which encodes a 45 amino acid polypeptide that allows auxin-induced protein degradation^34^, before the STOP codon of the endogenous *gsp-1*, *gsp-2*, and *hasp-1* loci was performed using overlapping single-stranded DNA (ssDNA) oligos (Supplementary Table 3) as repair templates and in vitro assembled Cas9 ribonucleoprotein complexes^57^. The following sgRNA sequences were used: TGGTAAGAAGTAAAGTGATG (*gsp-1*), ATCGACTGATGATTACTTCT (*gsp-2*), and AGCACATTATTGACGTGTCG (*hasp-1*).

To create a 1136 bp deletion at the endogenous *sgo-1* locus, two Cas9 ribonucleoprotein complexes targeting the 5´ (TGAAATTTATTGTTGGTTCA) and 3´ (CAGAAAAATGTATTGATGTA) ends of *sgo-1* were simultaneously injected to create 2 DSBs that were bridged together with a ssDNA oligo (Supplementary Table 3).

### Immunostaining and image acquisition

Germlines from 18 to 24 h post L4 hermaphrodites were dissected, fixed, and processed for immunostaining as described in ref. ^58^. Briefly, germlines were dissected in EGG buffer (118 mM NaCl, 48 mM KCl_2_, 2 mM CaCl_2_, 2 mM MgCl_2_, 5 mM HEPES) containing 0.1% Tween and immediately fixed in 1% paraformaldehyde for 5 min. Slides were frozen in liquid nitrogen, then immersed for at least 1 min in methanol at –20 °C and transferred to PBST (1× phosphate-buffered saline (PBS) and 0.1% Tween). Blocking in 0.5% bovine serum albumin in PBST was carried out for 1 h. Primary antibodies were incubated overnight at room temperature, slides were then washed 3 times for 5 min in PBST and secondary antibodies were added and incubated for 2 h at room temperature. Following 2 washes in PBST, the slides were counterstained with 4',6-diamidino-2-phenylindole (DAPI) and mounted using Vectashield. All images from immunostaining experiments were acquired with a Delta Vision system (Applied Precision) equipped with an Olympus 1× 70 microscope using a 100× lens. Images were subjected to deconvolution analysis using SoftWoRx 3.0 (Applied Precision) and images were mounted in Photoshop.

### Antibody production

Phospho-specific antibodies against S663 in REC-8 were produced by injecting rabbits with the synthetic peptide (QKSpSERLGVQFMRTDDC) (GenScript). Polyclonal pS663 antibodies were affinity purified by binding to a column containing the phospho-peptide. Specificity of the antibodies was validated by absence of staining in germlines of worms carrying the S663A mutation in REC-8. Phospho-specific antibodies against S11 in HTP-1 were produced as described above, but using the synthetic peptide (DEpSLNKSADSIDDKC) (GenScript). Specificity of the antibodies was validated by absence of staining in germlines of worms expressing a *htp-1*^*S11A*^ transgene in a *htp-1 (gk174), htp-2 (tm2543)* mutant background.

### Detection of phosphorylation sites by mass spectrometry

Large *C. elegans* cultures were prepared by seeding 20 100-mm NGM plates with 1 ml of OP50 bacteria (obtained from resuspending 2 liters of an overnight *E. coli* culture in a final volume of 40 ml). Between 5000 and 6000* C. elegans* embryos were added to each 100 mm plate, and the plates were incubated at 20 °C for 3 days. Young adult worms were collected and transferred to 50 ml tubes by washing the plates with M9, and tubes were left on a rack for 15 min to allow the worms to pellet by gravity, at which time most of the M9 was removed and fresh M9 solution was added. This washing step was repeated 3 times. The final wash was performed using NP buffer (10 mM HEPES-KOH pH 7.6, 1 mM EGTA, 10 mM KCl, 1.5 mM MgCl_2_, 0.25 mM Sucrose, 1 mM phenylmethylsulfonyl fluoride and 1 mM dithiothreitol) containing protease and phosphatase inhibitors and worms were pelleted by centrifugation at 600×*g* for 2 min. A total of 1 ml of this worm pellet was used to isolate nuclei. Worms were broken using a cooled metal Wheaton tissue grinder and the resulting worm solution was filtered first using a 100 μm mesh, followed by a second filtration with a 40 μm mesh. The filtered solution was then centrifuged at 300×*g* for two min at 4 °C, and the supernatant from this step, which contains nuclei, was further centrifuged at 2500×*g* for 10 min at 4 °C. The pellet from this step contained germline nuclei.

Normalized protein amounts from isolated nuclei were denatured and digested with trypsin enzyme. Peptides were desalted and then phosphopeptides were enriched through use of titanium dioxide. Phosphopeptides were separated by reverse-phase nano-high-performance liquid chromatography and analyzed by orbitrap and ion trap mass spectrometry (MS) to produce MS and MS/MS data. Files were processed using Mascot Distiller and searched against a WormPep protein database which was also reversed to produce a decoy database, enabling a false discovery rate (1%) to be calculated. Phosphorylation sites within peptides were localized and reported by Mascot delta score.

### Antibodies used for immunostaining

Primary antibodies (dilution indicated in brackets) were as follows: mouse anti-His tag (A00186-100; GenScript) (1:600), 488-conjugated rabbit anti-GFP (A21311; Molecular Probes) (1:200), mouse anti-FLAG (F1804; Sigma) (1:500), rabbit anti-LAB-1^24^, rabbit anti-AIR-2^24^, rabbit anti-P Aurora A/B/C (2914S; Cell Signaling); rabbit anti-H3 pS10 (16-189; Merck Millipore) (1:600), rabbit anti-H3 pT3 (07-424; Merck Millipore) (1:700), rabbit anti-ICP-1 (1:500), rabbit anti-REC-8 p663 (1:1000), and rabbit anti-HTP-1 S11 (1:200). All secondary antibodies were conjugated to Alexa-488 or Alexa-555 (Molecular Probes) and used at 1:500.

### Quantitative analysis of LAB-1 staining

Quantification of signal intensity was performed on unprocessed images acquired as three-dimensional stacks, and all genotypes were acquired using identical exposure settings on a Delta Vision system. The exposure settings on the Delta Vision system were adjusted to obtain a maximum value of 3600 of LAB-1 signal on the control strain that displayed the strongest LAB-1 signal (*htp-1::6His; htp-1Δ*). These exposure settings were then applied to all analyzed genotypes. For each bivalent and univalent analyzed, the intensity of LAB-1 staining was calculated as the difference between the maximum and minimum fluorescence intensity values. Then, this value was normalized across all genotypes by subtracting the average intensity measured in univalents of *htp1Δ htp-*2Δ double mutants, which were used as a negative control since LAB-1 is not loaded in the absence of HTP-1 and HTP-2.

### In vivo imaging of embryos

At 24 h post L4, hermaphrodites were dissected to release embryos in a drop of 60% v/v Leibowitz-15 media, 20% fetal bovine serum, 25 mM HEPES pH 7.4, and 5 mg/ml Inulin. Embryos were mounted as described in ref. ^59^ and imaged with a Delta vision system equipped with Olympus 1× 70 microscope. Images of the meiotic divisions were acquired as series of 1 μM-spaced *Z*-stacks (9–12 section) with a regular time lapse of 5 s intervals using a 60× lens. Videos of these time series were created using SoftWorx 3.0.

### Preparation of whole-worm protein extracts and western blotting

Whole-worm protein extracts were produced by picking 80 young hermaphrodites (24 h post L4) into a 1.5 ml Eppendorff tube containing 40 μl of 1× TE (10 mM Tris-HCl pH 8, 1 mM EDTA) supplied with complete protease inhibitor (Roche). The samples were subjected to three cycles of freezing/thawing using liquid nitrogen before Laemmli buffer was added to a 1× final concentration, and the samples were boiled for 10 min. Equal volumes of protein extracts were run on a 10% acrylamide gel (Bio-Rad) and transferred onto a nitrocellulose membrane for 1 h at 4 °C and then blocked for 1 h in 5% milk TBST (1% TBS and 0.1% Tween) at room temperature. Primary antibodies, mouse anti-His antibody (GenScript 0.2 μg/ml) and goat anti-Actin (Santa Cruz 1:3000), were diluted in 1× TBST and incubated overnight at 4 °C. The following secondary antibodies conjugated to horseradish peroxidase were used: goat anti-mouse (Jackson Immunoresearch) (1:5000) and donkey anti-goat (Sigma) (1:8000). Secondary antibodies were diluted in 1× TBST containing 5% milk and incubated for 1 h at room temperature.

### Scoring number of chromatin bodies in diakinesis oocytes

Worms were dissected and stained with DAPI as described in the immunostaining protocol and the number of DAPI-stained bodies present in the −1 oocyte was scored. All images were acquired using a Delta Vision Deconvolution system equipped with an Olympus 1× 70 microscope.

### Scoring number of polar bodies in embryos

Worms were dissected to release embryos, and then fixed and stained with DAPI as described in the immunostaining protocol. Polar bodies were counted in one- and two-cell stage embryos using a Delta Vision system equipped with an Olympus 1× 70 microscope.

### Screening of embryonic lethality and male progeny

L4 worms grown under physiological conditions were individually picked and transferred onto fresh plates every 12 h. The total number of embryos from each plate was counted after the mother was transferred to a new plate. The presence of dead embryos was assessed 12 h after the mother had been removed from each plate. The presence of male progeny was scored 2 days after the mother was removed from each plate.

### RNA interference

RNAi of *apc-2*, *icp-1*, *plk-1,* and *wee-1* was performed using clones from the Ahringer library. RNAi of *air-2* was performed by amplifying a 811 bp fragment of genomic DNA using the primers (5′-GTTTACCTGGCTCGCACAAA-3′ and 5′-AGCCTTGGGATCAACGACAA-3′). RNAi of *cdk-1* was performed by amplifying a 905 bp fragment of genomic DNA using the primers (5′-CGGAGTCGTCTACAAAGGCA-3′ and 5′-GCAGTATCGTCCAAAAGGTGC-3′). Both fragments were cloned into the pL440 vector with the Gateway system (Life Technologies) and transformed into HT115 (DE3) *E. coli*. All RNAi experiments were performed by feeding worms with HT115 bacteria transformed with a vector for IPTG-inducible expression of double-stranded RNA (dsRNA). Bacteria containing the desired vector, as well as empty vector (L4440) controls, were grown overnight at 37 °C in 20 ml of LB containing 50 µg/ml ampicillin. Cultures were then spun down and resuspended in 1 ml LB, before 100 µl of bacteria were seeded onto NGM agar plates containing 1 mM IPTG and 25 µg/ml ampicillin. Expression of dsRNA was induced by incubating the plates at 37 °C overnight. The day after, L4 worms were added onto the plates and incubated at 20 °C for 36–48 h, with the exception of *plk-1* RNAi experiments in which the incubation lasted 18 h.

PP1 RNAi was performed by microinjecting young adults with dsRNA corresponding to *gsp-1* and *gsp-2*. The dsRNA synthesis was performed using the protocol in ref. ^60^, and 24 h post injection, whole worms were fixed in 95% ethanol, let to air dry and stained with DAPI. Early embryos in uterus were imaged using a Delta Vision system.

### Auxin-mediated protein degradation

Depletion of proteins (GSP-1, GSP-2, or HASP-1) tagged with the AID sequence was performed by transferring worms expressing the desired tagged protein/s and carrying the *eSi38* transgene^34^ to NGM agar plates containing 4 mM auxin and OP50 bacteria. For data shown on Fig. 6a, at 24 h post L4, worms were incubated in 4 mM auxin plates for 3–7 h before dissection and immunostaining. For data shown in Fig. 6b, at 24 h post L4, worms were incubated in 4 mM auxin plates for 24 h before dissection and immunostaining.

### Data availability

The authors declare that all data supporting the findings of this study are available within the article and its Supplementary Information files. *C*. *elegans* strains and antibodies generated in this study are available from the corresponding author upon request.

## Electronic supplementary material

Supplementary Information

Description of Additional Supplementary Files

Supplementary Movie 1

Supplementary Movie 2

Supplementary Movie 3

Supplementary Movie 4
