# Author Correction: Spatiotemporal regulation of Aurora B recruitment ensures release of cohesion during *C. elegans* oocyte meiosis

**DOI:** 10.1038/s41467-018-05848-4

**Published:** 2018-08-29

**Authors:** Nuria Ferrandiz, Consuelo Barroso, Oana Telecan, Nan Shao, Hyun-Min Kim, Sarah Testori, Peter Faull, Pedro Cutillas, Ambrosius P. Snijders, Monica P. Colaiácovo, Enrique Martinez-Perez

**Affiliations:** 1 MRC London Institute of Medical Sciences, Imperial College London, London, W12 0NN UK; 2 Department of Genetics, Harvard Medical School, Boston, MA 02115 USA; 3 Present Address: Barts Cancer Institute, London, EC1M 6BQ UK; 4 Present Address: The Francis Crick Institute, London, NW1 1AT UK; 5 Present Address: SPST, Tianjin University, Tianjin, 300072, China

Correction to: *Nature Communications*; 10.1038/s41467-018-03229-5, published online 26 Feb 2018

The original version of this Article contained an error in the spelling of the author Ambrosius P. Snijders, which was incorrectly given as Ambrosious P. Snijders. This has now been corrected in both the PDF and HTML versions of the Article.

