# Supplementary material for: Spatiotemporal regulation of Aurora B recruitment ensures release of cohesion during C. elegans oocyte meiosis
Source: Nat Commun. 2018 Feb 26;9:834. doi: 10.1038/s41467-018-03229-5 (PMC5827026; doi:10.1038/s41467-018-03229-5)
Supplement: Supplementary file 1 — Supplementary Information [file 41467_2018_3229_MOESM1_ESM.pdf]

Supplementary Information

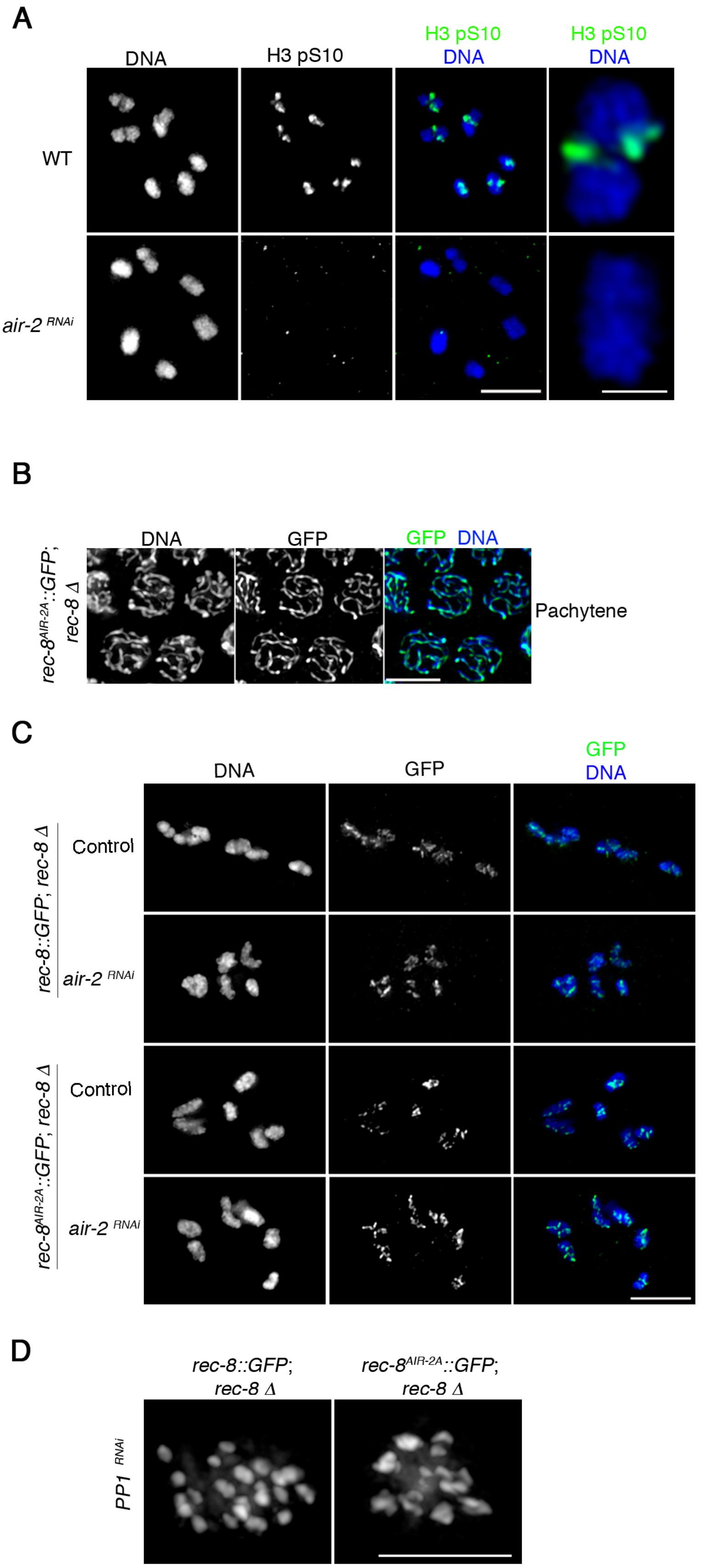

Supplementary Figure 1. A) Projections of diakinesis oocytes stained with anti-H3 pS10 antibodies and DAPI. AIR-2 knockdown by RNAi eliminates H3 pS10 staining. A single bivalent is magnified in the last column. **B)** Projections of late pachytene nuclei of *rec-8* $^{AIR\text{-}2A}$ mutants stained with anti-GFP antibodies and DAPI. GFP signal shows normal loading of REC-8 in pachytene and diakinesis nuclei. **C)** Diakinesis nuclei of indicated genotypes stained with anti-GFP antibodies and DAPI. Note that both REC-8::GFP and REC-8$^{AIR\text{-}2A}$::GFP localize to diakinesis bivalents. **D)** Projections of early embryos of indicated genotypes imaged in whole worms stained with DAPI following RNAi of PP1 (*gsp-1 + gsp-2*). Note gross separation of sister chromatids in *rec-8::GFP* (WT) embryos, but not in *rec-8*$^{AIR\text{-}2A}$ mutant embryos. The average number of DAPI-stained bodies observed following PP1 RNAi was 20.4 (range 19-21, n=9) in control embryos expressing REC-8::GFP and 9.8 (range 8-13, n= 9) in *rec-8*$^{AIR\text{-}2A}$ mutant embryos. Scale bars= 5 µm in all panels except single bivalent magnified in A, where scale bar= 1 µm..

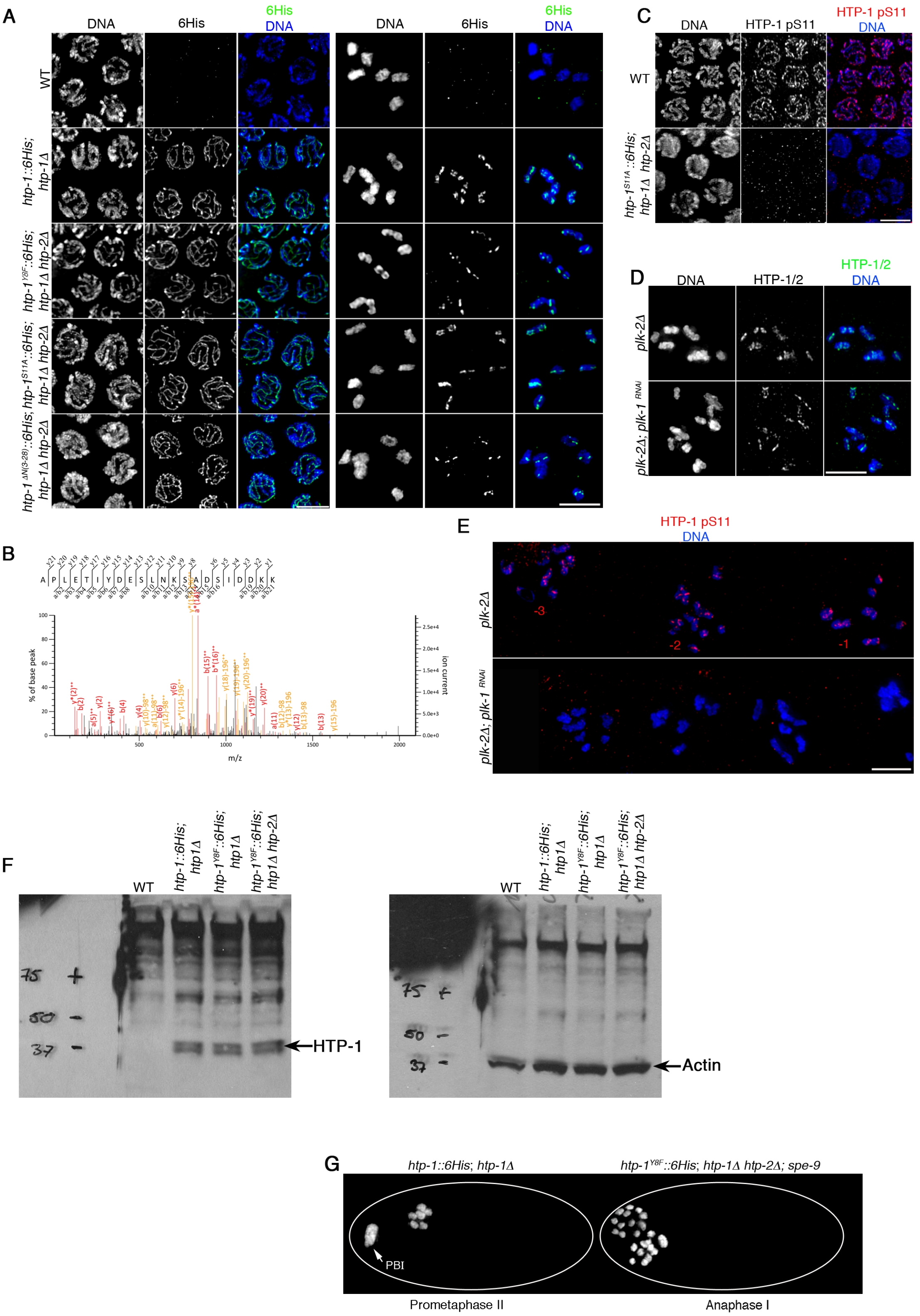

Supplementary Figure 2. A) Projections of pachytene nuclei and diakinesis oocytes of indicated genotypes stained with anti-6His antibodies and DAPI. WT (N2) worms were used as negative control. Note normal loading of HTP-1 in all

analysed genotypes. **B)**. MS/MS fragmentation spectrum for HTP-1 phosphopeptide APLETIYDESLNKSADSIDDKK in the range 100–2000/z. The annotated spectrum shows fragment ion species matched between theoretical and measured values. 'b ions' are generated through fragmentation of the peptide bond from the N-terminus, whereas 'y ions' are generated through fragmentation from the C-terminus. Ion species detected with a mass loss of 98 (1X phosphoric acid) or 196 (2X phosphoric acid) are indicated in yellow; those ions without phospho-loss are annotated in red. Note that the presence of several ions with a mass loss of 196 is consistent with the presence of two phosphorylated residues, and that the sites with the highest probability are S10 and S14, corresponding to S11 and S15 in HTP-1. **C)** Projections of pachytene nuclei stained with anti-HTP-1 pS11 antibodies and DAPI. Punctuate HTP-1 pS11 staining seen in WT nuclei is lost in *htp1*^{S11A}*::6His; htp-1Δ htp*-2Δ mutants. **D)** Projections of diakinesis oocytes stained with anti-HTP-1/2 antibodies and DAPI. HTP-1/2 signal is present in *plk-2 (tm1395) plk-1*^{RNAi} mutant oocytes. **E)** Projections of late diakinesis oocytes stained with anti-HTP-1 pS11 antibodies and DAPI. HTP-1 pS11 signal is absent in oocytes from *plk-2(tm1395) plk-1*^{RNAi} mutants, but not *plk-2(tm1395)* single mutants. **F)** Full blots used for panels shown in Fig 2I. **G)** Projections of DAPI-stained embryos. Embryo shown at the right was arrested at the end of anaphase I using the *spe-9(hc88)* ts allele. Note the presence of 2 groups of 12 chromatids in the *htp1*^{Y8F}*::6His; htp-1Δ htp*-2Δ mutant embryo, while the WT embryo contains a polar body and a group of 6 chromosomes. Scale bars= 5 µm.

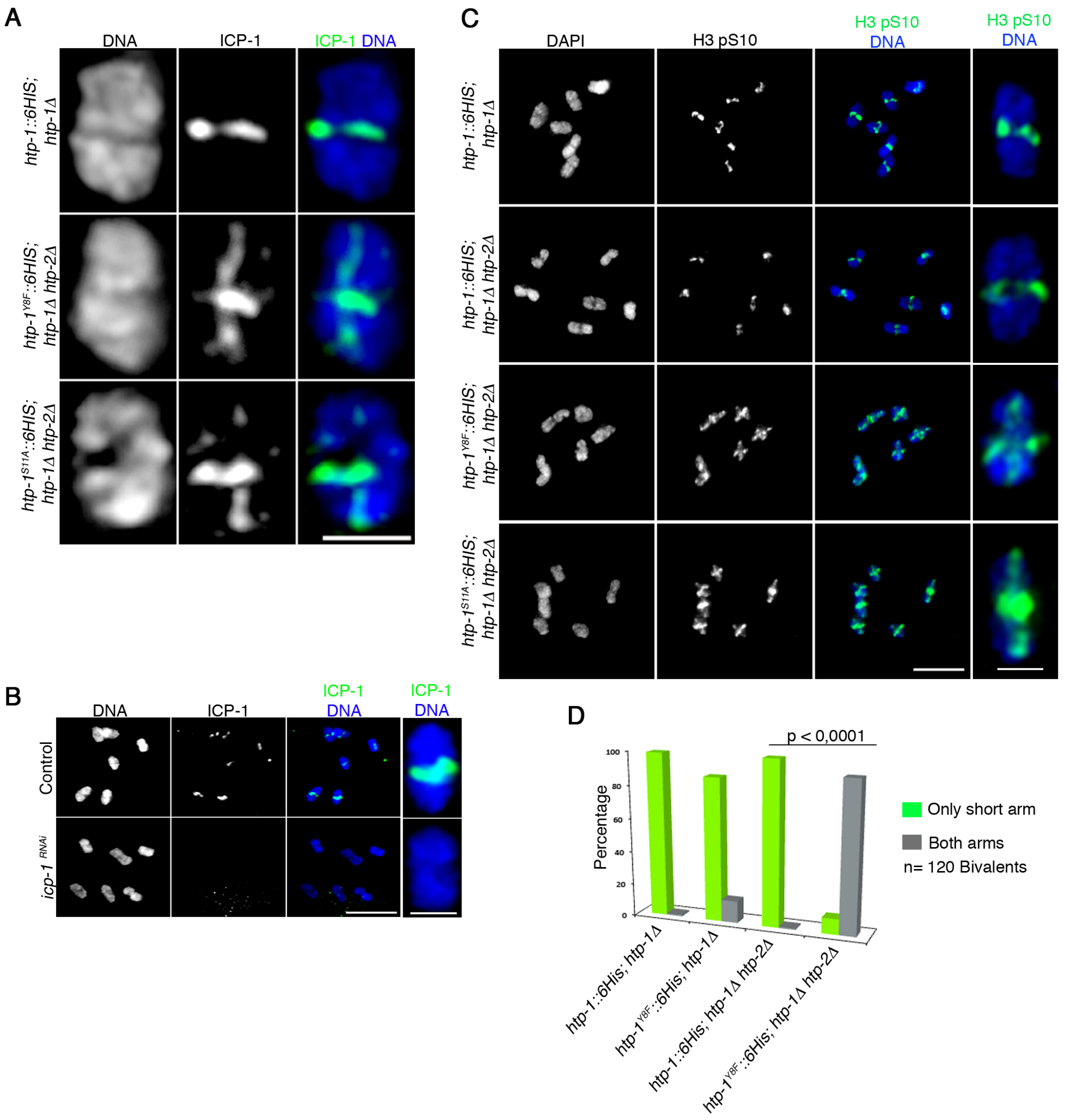

Supplementary Figure 3. A) Projections of single diakinesis bivalents stained with anti-ICP-1 antibodies and DAPI. ICP-1 staining is observed in the long arm when HTP-1^{S11A}::6His or HTP-1^{Y8F}::6His are expressed in a *htp1Δ htp*-2Δ double mutant background. **B)** Projections of diakinesis oocytes stained with anti-ICP-1 antibodies and DAPI. *icp-1* depleted oocytes lack ICP-1 signal, confirming the specificity of anti-ICP-1 antibodies. **C)** Projections of diakinesis oocytes stained with anti-H3 pS10 antibodies. H3 pS10 staining is present in the long and short arms when HTP-1^{S11A}::6His or HTP-1^{Y8F}::6His are expressed in a *htp1Δ htp*-2Δ double mutant background. **D)** Quantification of the number of diakinesis

bivalents displaying H3 pS10 signal only in the short arm (green bars) or in both arms (grey bars). p<0,0001 by chi-square test. Scale bars= 5 μm in whole oocyte projections and = 1 μm in panels containing a single magnified bivalent.

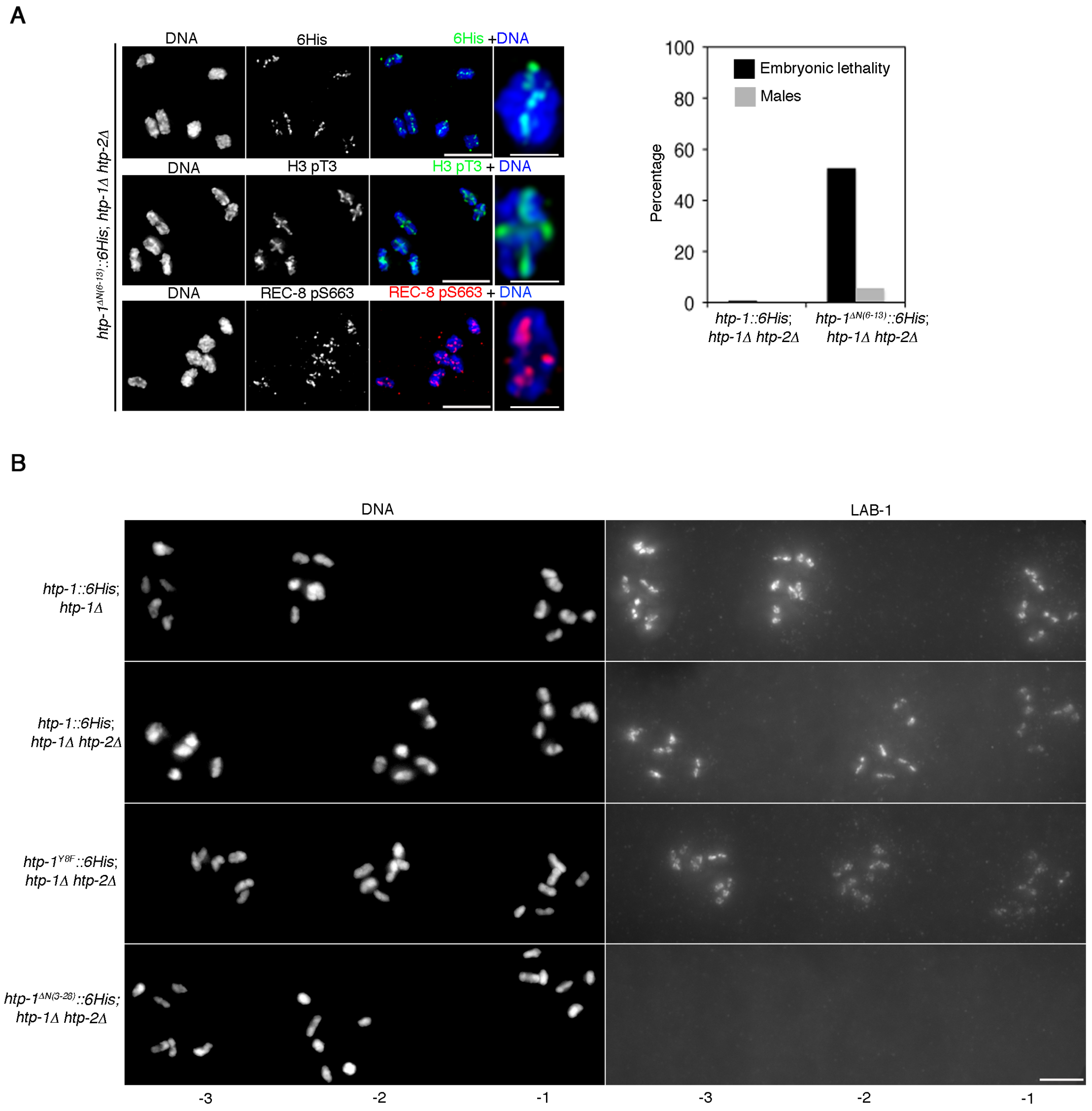

Supplementary Figure 4. (A) Projections of late diakinesis oocytes stained with anti-LAB-1 antibodies and DAPI. The position of the oocytes within the germline is indicated below the images. Data acquisition for all genotypes was performed using the same exposure settings and images shown are non-deconvolved projections adjusted with the same settings to allow visual comparisons of LAB-1 signal intensity. Note the reduction of LAB-1 signal intensity between the -2 and -1 oocytes of the top three genotypes. **B)** Percentage of embryonic lethality and incidence of male progeny in worms of indicated genotypes. Note high levels of embryonic lethality in *htp-1* $^{\Delta N(6\text{-}13)}$ *htp1Δ htp*-2Δ mutants. **C)** Projections of

diakinesis oocytes from *htp-1* $^{ΔN(6-13)}$; *htp1Δ htp*-2Δ mutants stained with anti-6His, anti-H3 pT3 and anti-REC-8 pS663 antibodies and DAPI. 6His staining (HTP-1) is limited to the long arm, while REC-8 pS663 and H3 pT3 signals are present in both the long and short arms. Scale bars= 5 µm in whole oocyte projections and = 1 µm in panels containing a single magnified bivalent.

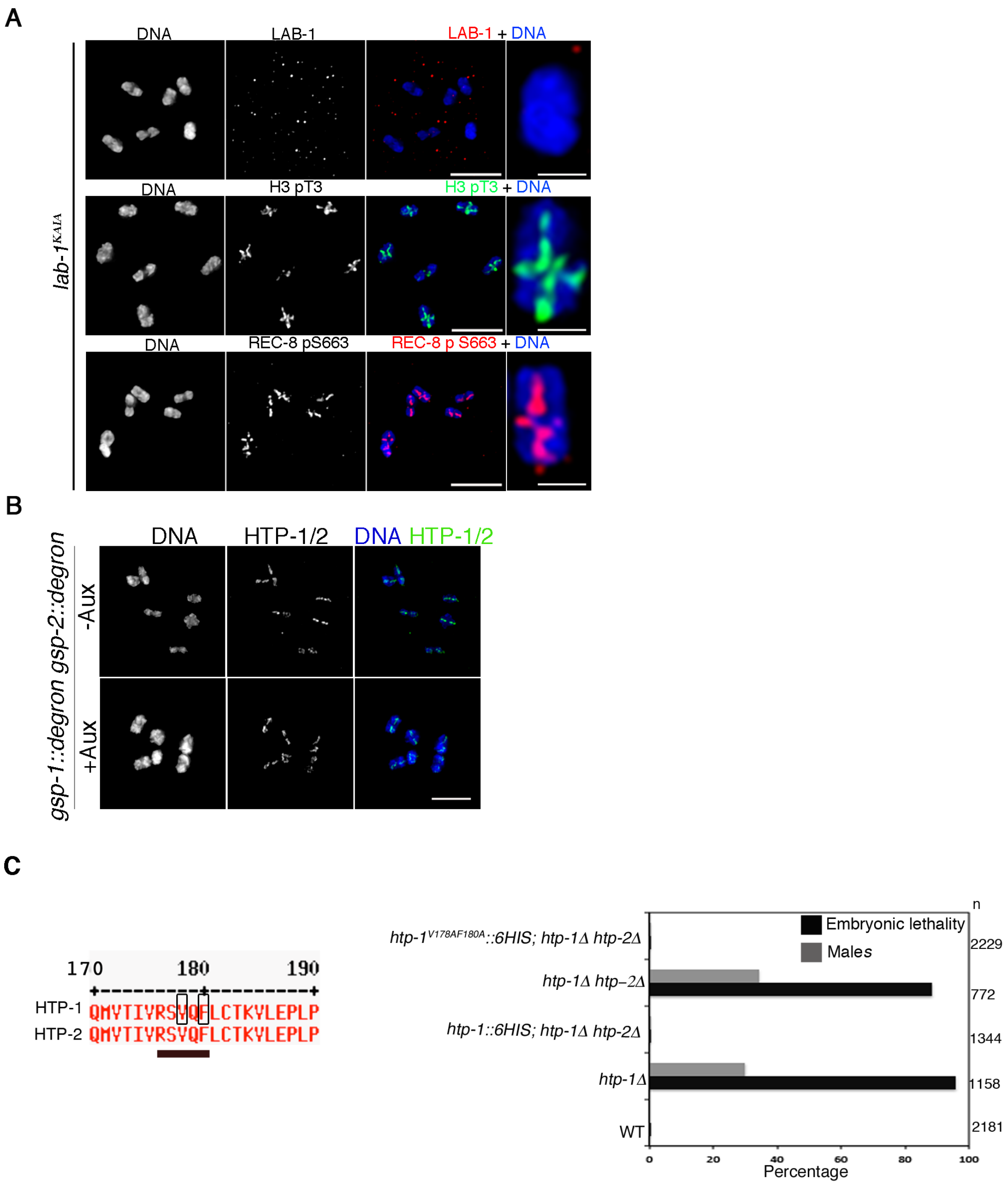

Supplementary Figure 5. A) Projections of late diakinesis oocytes from *lab-1*^{KAIA} mutants (*lab-1(rj46[V73A W75A])*) stained with anti-LAB-1, anti-H3 pT3, anti-REC-8 pS663 antibodies and DAPI. Note absence of LAB-1 signal and the presence of REC-8 pS663 and H3 pT3 staining in both the long and short arms. **B)** Projection of diakinesis oocytes stained with anti-HTP-1/2 antibodies and DAPI. Note that HTP-1/2 localize to the long arms of diakinesis bivalents following auxin-mediated depletion of PP1 (GSP-1 + GSP-2). **C)** Sequence

alignment of the HTP-1 and HTP-2 amino acid region containing a putative PP1 docking site (indicated by a horizontal line). Rectangles indicate amino acids mutated in the HTP-1$^{V178A\ F180A}$ mutants. Percentage of embryonic lethality and incidence of male progeny in worms of indicated genotypes. Note that expression of HTP-1$^{V178A\ F180A}$ in either *htp1Δ* single or *htp1Δ htp*-2Δ double mutant background does not induced embryonic lethality. Scale bars= 5 µm in whole oocyte projections and = 1 µm in panels containing a single magnified bivalent.

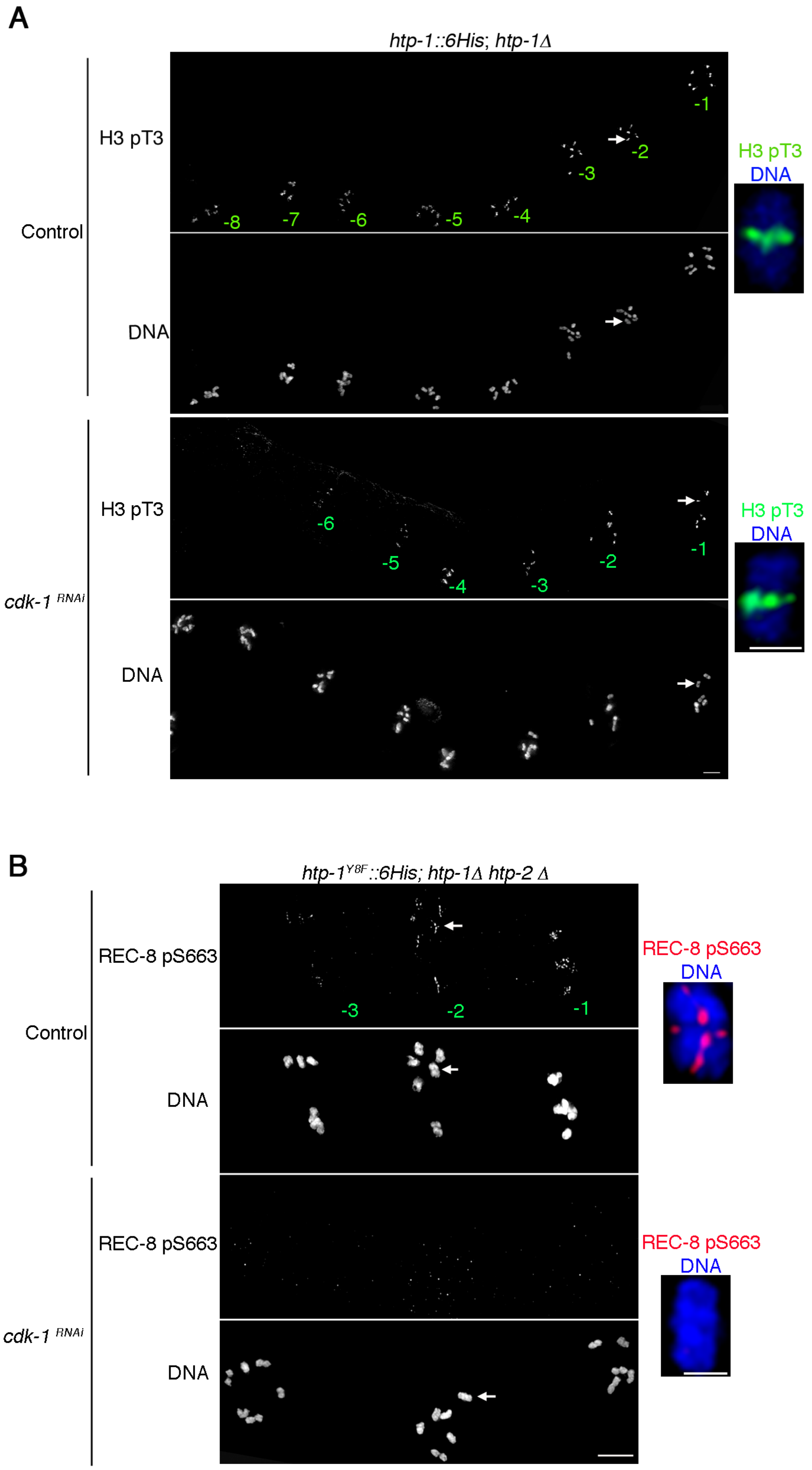

Supplementary Figure 6. A) Projections of diakinesis oocytes from *htp-1::6His; htp-1Δ* worms stained with anti-H3 pT3 antibodies and DAPI. Note that *cdk-1* depletion eliminates H3 pT3 staining in early diakinesis oocytes, but not in the -4,

-3, -2, and -1 oocytes. **B)** Projections diakinesis oocytes from *htp-1*^{Y8F}*::6His; htp1Δ htp*-2Δ mutants stained with anti-REC-8 pS663 antibodies and DAPI. REC-8 pS663 signal is absent in *cdk-1* depleted oocytes. Scale bars= 5 µm in whole oocyte projections and = 1 µm in panels containing a single magnified bivalent (indicated by arrows in panel containing all oocytes).

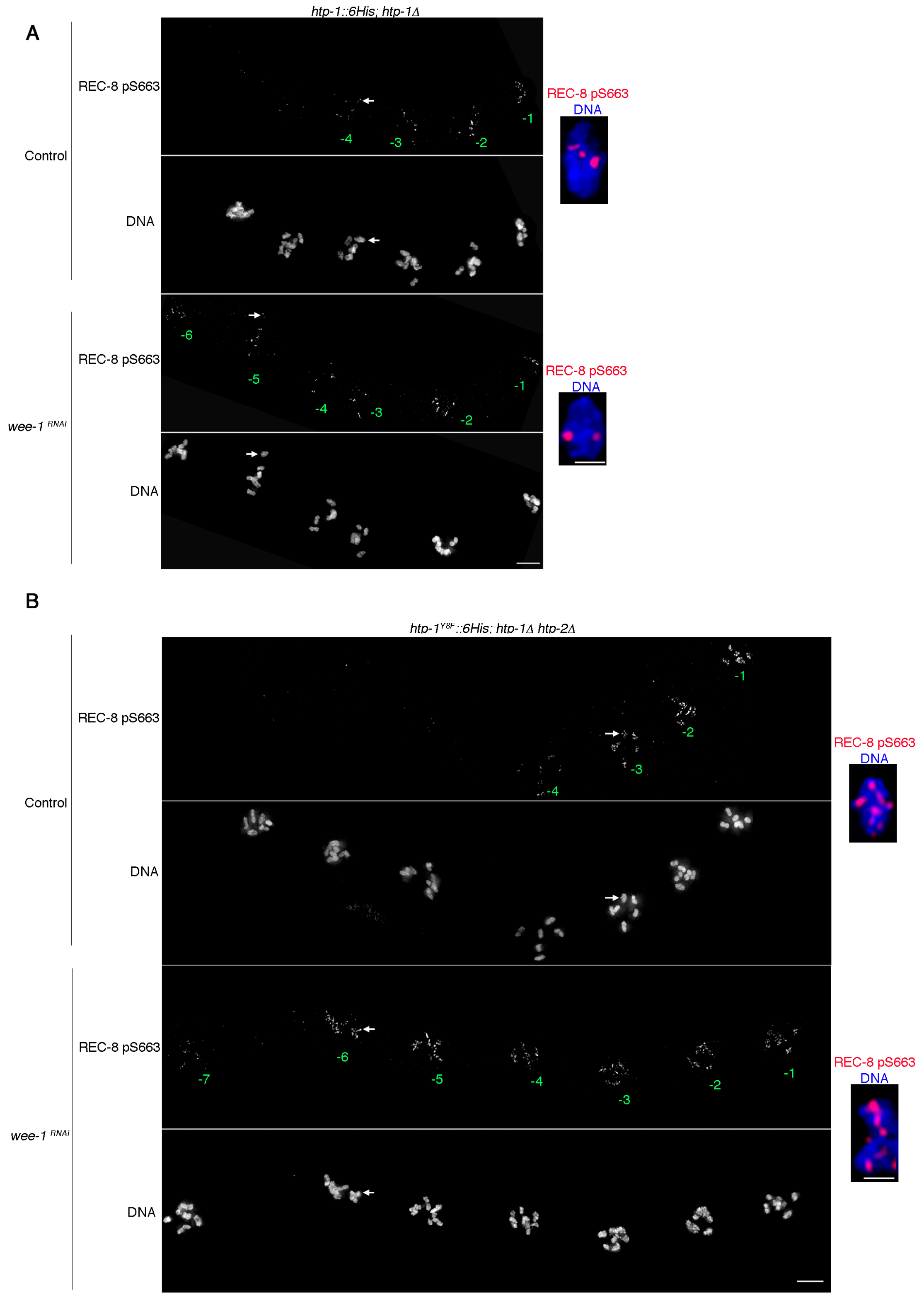

Supplementary Figure 7. A) Projections of diakinesis oocytes from *htp-1*::6His*; htp-1Δ* worms stained with anti-REC-8 pS663 antibodies and DAPI. REC-8 pS663 is present in the short arm of early diakinesis oocytes following depletion of *wee-*

1. **B)** Projections of diakinesis oocytes from *htp-1^{Y8F}::6His; htp1Δ htp*-2Δ mutants stained with anti-REC-8 pS663 antibodies and DAPI. REC-8 pS663 is present in both the short and long arms of early diakinesis oocytes following depletion of *wee-1*. Scale bars= 5 µm in whole oocyte projections and = 1 µm in panels containing a single magnified bivalent (indicated by arrows in panel containing all oocytes).

Mouse Rec8

```
        10         20         30         40         50
MFYYPNVLQR HTGCFATIWL AATRGSRLVK REYLNVNVVK TCEEILNYVL

        60         70         80         90        100
VRVQPPVAGL PRPRFSLYLS AQLQIGVIRV YFQQCQYLVE DIQHILEHLH

       110        120        130        140        150
RAQLRIRIDM EEADLPSLLL PNCLAMMETL EDAPEPFFGK MSVDPRLPSP

       160        170        180        190        200
FDIPQIRHLL EAATPEKTRK ETLPEATPDP RKPDRTLATV QSPEVITLQE

       210        220        230        240        250
AEPIRMLQIE GEQDLPEISR GDLELLIAEK DDAILLEERQ RGRLLRQRRA

       260        270        280        290        300
SLPLDESREE PRALEGAGLV SALSPPAPAQ VEGIQEALPG QVFPPEVQKM

       310        320        330        340        350
TGWEPGALLT EVTPPQELRL PAPPSTEKRL PSLQRPLPRR HRRRQLLFWD

       360        370        380        390        400
KETQISREKF EEQLQTGAHC WEYPVAQPPK RMLTSPAELF RTPTLSGWLP

       410        420        430        440        450
PELLGLWTHC AQVPQRMLRQ RPQLETEETV EEERAADEEE RRKTEALSEI

       460        470        480        490        500
EVLREAQEPS GPLMLSSELS LEAAEDEKSR TSLIPPEWWA WSEEGQPEPP

       510        520        530        540        550
ALPMLPELPE VPMEMPPRPE LSSEAVLRAV ALKLQANKEL DFSSLVPPLS

       560        570        580        590
PRKLASRVFY LLLVLSTQKI LLVEQQKPYG PLLIRPGPKF P
```

▼ AIR-2 consensus sites (R/KxS/T)

▭ Separase consensus sites (ExxR)

Supplementary Figure 8. Amino acid sequence of mouse Rec8 indicating the position of consensus motifs for AIR-2 phosphorylation (R/KXS/T) (triangles) and separase (EXXR) (boxes).

Transgene	Genotype	Origin
fqsi23	[*Prec-8 rec-8* ::*GFP 3'UTR rec*-8; *cb-unc-119(+)*]	[1]
fqSi417	[*Prec-8 rec-8* $^{AIR-2A}$ ::*GFP 3'UTR rec*-8; *cb-unc-119(+)*]	This study
fqSi22	[*Phtp-1 htp-1*::6His *3'UTR htp-1*; *cb-unc-119(+)*]	[2]
fqSi147	[*Phtp-1 htp-1* ^{Y8F}*::6His 3'UTR htp-1*; *cb-unc-119(+)]*	This study
fqSi240	[*Phtp-1 htp-1* ^{S11A}*::6HIS 3'UTR htp-1* ; cb-unc-119(+)]	This study
fqSi251	[*Phtp-1 htp-1* ^{S15A}::*6His 3'UTR htp-1; cb-unc-119(+)]*	This study
fqSi297	[*Phtp-1 htp-1*$^{\Delta N(3-28)}$*::6His 3'UTR htp-1; cb-unc-119(+)]*	This study
fqSi300	[*Phtp-1 htp-1*$^{\Delta N(6-13)}$*::6His 3'UTR htp-1; cb-unc-119(+)*]	This study
fqSi267	[*Phtp-1 htp-1*$^{V178A\ F180A}$::*6His 3'UTR htp-1; cb-unc-119(+)]*	This study
fqSi21	[*Phtp-2 htp-2::FLAG 3'UTR htp-2; cb-unc-119(+)*]	This study
fqSi210	[*Phtp-2 htp-2* ^{Y8F}*::FLAG 3'UTR htp-2; cb-unc-119(+)*]	This study
ojls50	[*Ppie-1p::GFP::air-2 unc-119(+)*]	[3]
weSi14	[*Pmex-5::mCherry::(Gly)5 Ala/his-58/tbb-2 3'UTR; cb-unc-19(+)*]	[4]
ieSi38	*[Psun-1::TIR1::mRuby::sun-1 3'UTR, cb-unc-119(+)]*	[5]

Supplementary Table 1: List of transgenes used in this study.

Strain	Genotype
ATGSi23	*fqSi23 II ; rec-8(ok978) IV*
ATGSi422	*fqSi417 II ; rec-8(ok978) IV / nT1 [unc-? (n754) let-? qls50] (IV;V)*
ATGSi25	*fqSi22 II ; htp-1(gk174) IV*
ATGSi195	*fqSi22 II ; htp-1(gk174) htp-2(tm2543) IV*
ATGSi155	*fqSi147 II ; htp-1(gk174) IV*
ATGSi159	*fqSi147 II ; htp-1(gk174) htp-2(tm2543) IV / nT1 [unc-? (n754) let-? qls50] (IV;V)*
ATGSi243	*fqSi240 II ; htp-1(gk174) IV*
ATGSi249	*fqSi240 II ; htp-1(gk174) IV htp-2(tm2543) V / nT1 [unc-? (n754) let-? qls50] (IV;V)*
ATGSi256	*fqSi251 II ; htp-1(gk174) IV ; htp-2(tm2543) V*
ATGSi294	*fqSi297 II ; htp-1(gk174) IV*
ATGSi386	*fqSi297 II ; htp-1(gk174) htp-2(tm2543) IV / nT1 [unc-? (n754) let-? qls50] (IV;V)*
ATGSi388	*fqSi300 II ; htp-1(gk174) htp-2(tm2543) IV / nT1 [unc-? (n754) let-? qls50] (IV;V)*
ATGSi273	*fqSi267 II ; htp-1(gk174) htp-2(tm2543) IV*
ATGSi196	*spe-9(hc88) I; fqSi147 II ; htp-1(gk174) htp-2(tm2543) IV / nT1 [unc-? (n754) let-? qls50] (IV;V)*
ATGSi163	*fqSi21 IV ; htp-2(tm2543) IV*
ATGSi232	*fqSi210 htp-1(gk174) htp-2(tm2543) IV / nT1 [unc-? (n754) let-? qls50] (IV;V)*
ATGSi241	*fqSi147 II ; fqSi210 htp-1(gk174) htp-2(tm2543) IV / nT1 [unc-? (n754) let-? qls50] (IV;V)*
ATG258	*lab-1(rj46) I /hT2 [bli-4(e937) let-?(q782) qIs48] (I;III)*
ATG357	*gsp-1(fq51 [gsp-1::degron]) V; gsp-2(fq49[gsp-2::degron]) III; ieSi38 [Psun-1::TIR1::mRuby::sun-1 3'UTR, cb-unc-119(+)] IV*
ATG385	*hasp-1(fq52 [hasp-1::degron]) I; ieSi38 [Psun-1::TIR1::mRuby::sun-1 3'UTR, cb-unc-119(+)] IV*
ATG354	*sgo-1(fq48) IV*

Supplementary Table 2. List of strains created in this study.

Supplementary Table 3. ssDNA repair oligos used in this study

sgo-1 deletion

gtttatgtgtaaaatacaaatgtttcagTCCCTGATTGATACCCATACGATGTTCCAGATTACGCT
ATCAATACATTTTTCTGAccaatttcattgaagat

hasp-1::degron

Left oligo:
CGATTTTTACGAGGGACCGATCGGAATGTCCACGACGAGACAGatgcctaaagatccagcca
aacctccggccaaggcacaagttgtgggatggccaccggtgagatcataccggaagaac

Right oligo:
tgggatggccaccggtgagatcataccggaagaacgtgatggtttcctgccaaaaatcaagcggtggcccggaggc
ggcggcgttcgtgaagTAAtgtgctcagaatatgagtatcaaaaaaatccg

gsp-1::degron

Left oligo:
GCGGAGGACGGCCTGGAACGACTGCTGGTAAGAAGatgcctaaagatccagccaaacctccggc
caaggcacaagttgtgggatggccaccggtgagatcataccggaagaac

Right oligo:
tgggatggccaccggtgagatcataccggaagaacgtgatggtttcctgccaaaaatcaagcggtggcccggaggc
ggcggcgttcgtgaagTGAagtgatgaggagaaagaacatttttgcctacatt

gsp-2::degron

Left oligo:
CGCACCGGCTGCGCAACCAAAGAAGGGTGCCAAGAAGatgcctaaagatccagccaaacctccg
gccaaggcacaagttgtgggatggccaccggtgagatcataccggaagaac

Right oligo:
tgggatggccaccggtgagatcataccggaagaacgtgatggtttcctgccaaaaatcaagcggtggcccggaggc
ggcggcgttcgtgaagTAAtcatcagtcgatttatctatcatctgtaaata

Supplementary References

1 Crawley, O. *et al.* Cohesin-interacting protein WAPL-1 regulates meiotic chromosome structure and cohesion by antagonizing specific cohesin complexes. *Elife* **5**, e10851, doi:10.7554/eLife.10851 (2016).

2 Silva, N. *et al.* The fidelity of synaptonemal complex assembly is regulated by a signaling mechanism that controls early meiotic progression. *Dev Cell* **31**, 503-511, doi:10.1016/j.devcel.2014.10.001 (2014).

3 Heallen, T. R., Adams, H. P., Furuta, T., Verbrugghe, K. J. & Schumacher, J. M. An Afg2/Spaf-related Cdc48-like AAA ATPase regulates the stability and activity of the C. elegans Aurora B kinase AIR-2. *Dev Cell* **15**, 603-616, doi:10.1016/j.devcel.2008.08.005 (2008).

4 Zeiser, E., Frokjaer-Jensen, C., Jorgensen, E. & Ahringer, J. MosSCI and gateway compatible plasmid toolkit for constitutive and inducible expression of transgenes in the C. elegans germline. *PLoS One* **6**, e20082, doi:10.1371/journal.pone.0020082 (2011).

5 Zhang, L., Ward, J. D., Cheng, Z. & Dernburg, A. F. The auxin-inducible degradation (AID) system enables versatile conditional protein depletion in C. elegans. *Development* **142**, 4374-4384, doi:10.1242/dev.129635 (2015).
