# Supplementary material for: Spatiotemporal regulation of Aurora B recruitment ensures release of cohesion during C. elegans oocyte meiosis
Source: Nat Commun. 2018 Feb 26;9:834. doi: 10.1038/s41467-018-03229-5 (PMC5827026; doi:10.1038/s41467-018-03229-5)
Supplement: Supplementary file 2 — Description of Additional Supplementary Files [file 41467_2018_3229_MOESM2_ESM.pdf]

Description of Additional Supplementary Files

File Name: Supplementary Movie 1

Description: Meiotic divisions in a WT embryo expressing histone H2B::mcherry. Filming covers the interval between metaphase I and the first mitotic division. The first meiotic division produces a polar body and a nucleus containing 6 chromosomes, which undergoes the second meiotic division producing a second polar body plus the oocyte pronucleus. The movie also shows fusion of the oocyte and sperm pronucleous and the first mitotic division of the embryo. Individual frames form this movie are shown in Figure 1H and Figure 2K.

File Name: Supplementary Movie 2

Description: Meiotic divisions in a rec-8AIR-2A mutant (rec-8 AIR-2A::GFP; rec-8Δ) embryo expressing histone H2B::mcherry. Filming covers the interval between metaphase I and the first mitotic division. Note the presence of chromatin threads between the two groups of separating chromosomes during the first meiotic division. The second meiotic division produces an oocyte pronucleus that appears to contain less chromatin than the second polar body, suggesting unequal chromosome segregation during anaphase II. Individual frames form this movie are shown in Figure 1H.

File Name: Supplementary Movie 3

Description: Meiotic divisions in a rec-8 mutant (rec-8Δ) embryo expressing histone H2B::mcherry. Filming covers the interval between metaphase I and the first mitotic division. The first meiotic division produces a polar body and a prophase II nucleus that eventually undergoes chromosome decondensation, without a second round of chromosome segregation and fuses with the sperm pronucleous.

File Name: Supplementary Movie 4

Description: Meiotic divisions in a htp-1Y8F::6His (htp-1Δ htp-2Δ) mutant embryo expressing histone H2B::mcherry. Filming covers the interval between metaphase I and the first mitotic division. The first meiotic division produces a polar body and a nucleus in which up to twelve chromosomes can be observed. A second round of chromosome segregation does not take place. The nucleus containing twelve chromosomes fuses with the sperm pronucleous. Individual frames form this movie are shown in Figure 2K.
